# Nuclear Argonaute:miRNA complexes recognize target sequences within chromatin-associated RNA and silence gene expression

**DOI:** 10.1093/nar/gkaf800

**Published:** 2025-08-27

**Authors:** Cristina R Hofman, Jiaxin Hu, Rut Bryl, Victor Tse, David R Corey

**Affiliations:** UT Southwestern Medical Center, Department of Pharmacology and Biochemistry, 6001 Forest Park Road, Dallas TX 75390, United States; UT Southwestern Medical Center, Department of Pharmacology and Biochemistry, 6001 Forest Park Road, Dallas TX 75390, United States; UT Southwestern Medical Center, Department of Pharmacology and Biochemistry, 6001 Forest Park Road, Dallas TX 75390, United States; UT Southwestern Medical Center, Department of Pharmacology and Biochemistry, 6001 Forest Park Road, Dallas TX 75390, United States; UT Southwestern Medical Center, Department of Pharmacology and Biochemistry, 6001 Forest Park Road, Dallas TX 75390, United States

## Abstract

The action of microRNAs (miRNAs) in mammalian cells involves recognition of messenger RNA (mRNA) in the cytoplasm and inhibition of translation. Both miRNAs and their associated protein factors, however, are present in mammalian cell nuclei. It is unclear how this nuclear localization affects endogenous gene expression. Here, we use chimeric eCLIP to identify complexes of Argonaute 2 (AGO2) and miRNAs. We identify the most abundant miRNAs associated with chromatin and their chromatin-associated RNA targets. Chimeric eCLIP revealed that high mobility group AT-Hook 2 (*HMGA2*) was the most compelling target for miRNA-mediated gene regulation. There are four confirmed *let-7* miRNA sites within the 3′-UTR in the cytoplasm or nucleus and three within chromatin-associated RNA. The expression of mature *HMGA2* mRNA was repressed by *let-7* in both the cytoplasm and the nucleus. *let-7* had little effect on *HMGA2* transcription or splicing. Our data validate chimeric eCLIP as a powerful method for experimentally identifying promising miRNA:RNA interactions. Rather than a solely cytoplasmic event, binding of miRNA-associated protein factors to mRNA targets may begin in the nucleus. Gene silencing reduces RNA levels in both the cytoplasm and the nucleus. miRNA-mediated silencing of mRNAs may be influenced by both nuclear and cytoplasmic interactions.

## Graphical Abstract

**Graphical Abstract:**
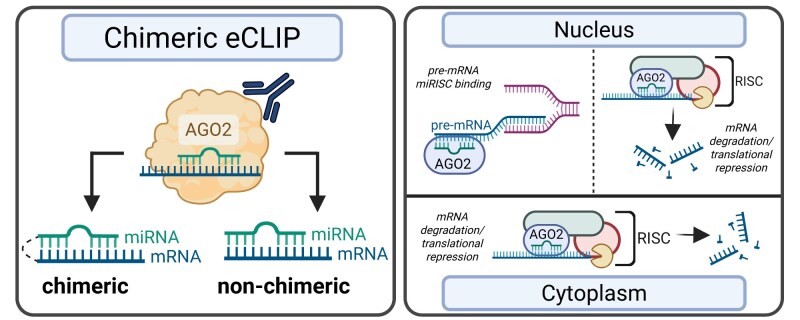

## Introduction

MicroRNAs (miRNAs) are key components of a versatile mechanism for gene regulation [1–3]. Argonaute (AGO) protein is loaded with a microRNA (miRNA) or synthetic duplex RNA to form the miRNA-induced silencing complex (miRISC). In the cytoplasm, miRISC acts as a programmable regulatory factor, with the small RNA facilitating recognition of an RNA target by Watson–Crick base pairing. The AGO protein protects the small RNA, promotes binding to target messenger RNA (mRNA), and silences these targets through either enzymatic cleavage or recruitment of additional proteins for translational repression and transcript degradation [4–7]. The diversity of synthetic RNAs is essentially infinite and there are hundreds of well validated miRNAs in human cells, suggesting the potential to target almost any accessible cellular RNA.

In mammalian cells, regulation by miRNAs is generally associated with the silencing of genes in the cytoplasm. However, miRNAs [8] and protein factors like AGO proteins [9–12] are also present and functional in mammalian cell nuclei. Exogenous synthetic RNAs have been shown to modulate transcription [13–18] and splicing [19–21]. Endogenous miRNAs have been found to control transcription [14, 22–24], splicing [25, 26], silence mobile transposons in quiescent cells [27], and influence gene expression in spermatogenic cells through association with meiotic chromatin [28]. In other organisms, including *Schizosaccharomyces pombe* and *Arabidopsis thaliana*, endogenous RISC has roles in regulation of chromatin dynamics [29–31] in addition to its role in transcriptional regulation. While these studies suggest the potential for diverse and robust nuclear regulation in mammalian cells, the full biological impact of nuclear miRNAs and their potential for modulating gene expression remains to be understood.

While miRNAs have hundreds of potential targets inside human cells [32] and may produce many statistically significant effects on gene expression, target identification is rarely a simple task [33, 34]. Our previous work found that AGO binding in a gene 3′-untranslated region (3′-UTR) alone does not always lead to repression [35]. Other studies have shown that many individual miRNAs are not essential for viability or development [36, 37]. These findings suggest that commonly used methods of target identification, including genetic manipulation of miRNAs or CLIP to identify AGO binding sites, may produce false positive results, fail to identify promising candidates, and may not completely consider the biology underlying the interactions. While bioinformatic target predictions can be used to identify seed sequence complementarity, analysis must take into account evolving definitions about the identity of functional miRNAs [33, 38–40]. Ultimately, the identity and impact of regulatory miRNAs likely varies among cell types and finding the biologically significant miRNA:target interactions among the many potential interactions can be like finding a needle in a haystack [41].

Fortunately, contemporary experimental tools allow higher confidence identification of some miRNA:target interactions. While previous work largely relied on genetic manipulation and microarrays [36, 37, 42], advances in crosslinking and immunoprecipitation (CLIP) allow us to identify experimentally identify AGO2 binding sites [43–45]. More specialized CLIP techniques even allow for the identification of specific miRNA:mRNA interactions [46, 47]. While beneficial advances, these techniques have limitations and offer no guarantee that they will identify all biologically relevant control points for miRNAs. However, in tandem, they offer powerful tools for prioritizing potential miRNA targets for experimental validation.

In this report, we use chimeric Enhanced Crosslinking Immunoprecipitation (eCLIP) [48] to obtain complementary datasets that define potential miRNA targets in HCT116 human colorectal cancer cells. These data identify high mobility group AT-Hook 2 (*HMGA2*) as the best candidate for miRNA-mediated regulation. We demonstrate that recognition of the *HMGA2* 3′-untranslated region begins when the RNA is associated with chromatin and can also be detected throughout cell nuclei and the cytoplasm. Gene silencing can be observed in the nucleus. For *HMGA2*, however, changes in transcription and splicing are not observed. These data suggest that the journey of miRNA-mediated recognition and silencing can begin in mammalian cell nuclei and chromatin.

## Materials and methods

### Cell culture

Wild-type HCT116, *DROSHA*^−/−^, NLS–AGO2, and HT-29 cells were cultured in McCoy’s 5A medium supplemented with 10% FBS. HeLa cells were grown in Dulbecco’s Modified Eagle’s medium (DMEM) supplemented with 10% FBS. All cells were grown at 37°C in 5% CO_2_ and passed when 70%–80% confluent.

### Preparation of cytoplasm, nucleus, nucleoplasm, and chromatin extracts

Cells were seeded at 2.5 × 10^6^ cells per dish into 150 mm dishes and supplemented with 60 ml of fresh media on day 2 after seeding. Cells were harvested 3 days after seeding with trypsin. Subcellular fractionation to isolate cytoplasm and nucleus extracts were as previously described and isolation of cytoplasm, nucleoplasm, and chromatin extracts was as previously described with modifications [11, 49, 50]. Samples were kept on ice and ice-cold buffers were used. Briefly, harvested cells were pelleted and washed with Phosphate Buffered Saline (PBS). Cells were pelleted and lysed with hypotonic lysis buffer (HLB) (10 mM Tris–HCl pH 7.4, 10 mM NaCl, 3 mM MgCl_2_, 2.5% NP-40, and 0.5 mM dithiothreitol (DTT)). Nuclei were pelleted and the supernatant was collected as the cytoplasmic fraction. Nuclei were washed a total of six times with HLB. After washing, the nuclei were lysed with modified Wuarin-Schibler buffer (MWS) (10 mM Tris–HCl pH 7.0, 4 mM EDTA, 0.3 M NaCl, 1 M urea, and 1% NP-40). Chromatin was pelleted and supernatant was collected as the nucleoplasm fraction. Chromatin was washed twice with MWS and lysed by sonication in lysis buffer (50 mM Tris–HCl pH 7.4, 120 mM NaCl, 0.05% NP-40, 1 mM EDTA, and 1 mM DTT).

### Western blot analysis

Proteins were separated on 4%–20% gradient Mini-PROTEAN^®^ TGXTM precast gels (Bio-Rad). After gel electrophoresis, proteins were wet transferred to nitrocellulose membrane (0.45 μM, GE Healthcare Life Sciences) at 100 V for 90 min. Membranes were blocked for 1 h at room temperature with 5% milk in 1× PBS containing 0.05% Tween-20. Blocked membranes were incubated with the primary antibodies in blocking buffer at 4°C on rocking platform overnight: anti-AGO1 1:1000 (Cell Signaling, #5053), anti-AGO2 1:2000 (Fujifilm Wako, 015-22031), anti-AGO3 1:1000 (Cell Signaling, #5054), anti-AGO4 1:1000 (Cell Signaling, #6913), anti-TNRC6A 1:5000 (A302-329A, Bethyl), anti-Drosha 1:500 (Cell Signaling, #3364), anti-Dicer (Cell Signaling, #3363), anti-Calnexin 1:5000 (Cell Signaling, #2433), anti-β-tubulin 1:5000 (Sigma–Aldrich, T5201), SP1 1:5000 (Cell Signaling, #5931), and Histone H3 1:20K (Cell Signaling, #2650). After primary antibody incubation, membranes were washed 3× for 10 min at room temperature with 1× PBS + 0.05% Tween-20 (PBST 0.05%) and then incubated for 1 h at room temperature with respective secondary antibodies in blocking buffer. Membranes were washed again 3× 15 min in PBST 0.05%. Washed membranes were soaked with HRP substrate according to manufacturer’s recommendations (SuperSignal^™^ West Pico Chemiluminescent substrate, Thermo Scientific) and exposed to films. The films were scanned, and bands were quantified using ImageJ software.

### Chimeric eCLIP (miR-eCLIP)

Cells were seeded in 150 mm dishes and grown until 80%–90% confluent. Cells were then UV-crosslinked on ice at 254 nm (400 mJ/cm^2^). Cells were collected or fractionated as described above. Prior to storage, cell pellets and lysates were flash frozen.

For miR-eCLIP experiments, the standard eCLIP protocol [45] was modified to enable chimeric ligation of miRNA and mRNA [48]. Studies were performed by Eclipse Bioinnovations Inc. (San Diego, www.eclipsebio.com). Cells were then lysed in 1000 μl of eCLIP lysis mix and sonicated (QSonica Q800R2) for 5 min, 30 s on/30 s off with an energy setting of 75% amplitude. The chromatin fractions from UV crosslinked cells were lysed in 1000 μl of eCLIP lysis mix and sonicated (QSonica Q800R2) for 17 min, 30 s on/30 s off with an energy setting of 75% amplitude. The cytoplasmic fractions from UV crosslinked cells were sonicated (QSonica Q800R2) for 5 min, 30 s on/30 s off with an energy setting of 75% amplitude and diluted in 2× volume of 1.5× eCLIP lysis buffer. Equivalent amounts of sample lysates were digested with RNase-I (Ambion). A primary mouse monoclonal AGO2/EIF2C2 antibody (sc-53521, Santa Cruz Biotechnology) was incubated for 1 h with magnetic beads pre-coupled to the secondary antibody (M-280 Sheep Anti-Mouse IgG Dynabeads, Thermo Fisher 11202D) and added to the homogenized lysate for overnight immunoprecipitation at 4°C. Following overnight IP, 2% of the sample was taken as the paired size-matched input with the remainder magnetically separated and washed with eCLIP high stringency wash buffers. The chromatin samples were subjected to DNase treatment (TURBO DNase Thermo Fisher) followed by eCLIP high stringency wash step. Chimeric ligation was then performed on-bead at room temperature for 1 h with T4 RNA ligase (NEB). IP samples were then dephosphorylated with alkaline phosphatase (FastAP, Thermo Fisher) and T4 PNK (NEB), and an RNA adapter was ligated to the 3′ ends. IP and input samples were cut from the membrane at the AGO2 protein band size to 75 kDa above. Western blot was visualized using anti-AGO2 primary antibody (50683-RP02, SinoBiological) at a 1:4000 dilution, with TrueBlot anti-rabbit secondary antibody (18-8816-31, Rockland) at 1:8000 dilution. RNA adapter ligation, reverse transcription, DNA adapter ligation, and PCR amplification were performed as previously described.

After sequencing, samples were processed with Eclipsebio’s proprietary analysis pipeline (v1). Unique molecular identifiers (UMIs) were pruned from read sequences using umi_tools (v1.1.1). Next, 3′ adapters were trimmed from reads using cutadapt (v3.2). Reads were then mapped to a custom database of repetitive elements and rRNA sequences. All nonrepeat mapped reads were mapped to the hg38 genome using STAR (v2.7.7a). PCR duplicates were removed using umi_tools (v1.1.1). AGO2 eCLIP peaks were identified within eCLIP samples using the peak caller CLIPper (v2.0.1). For each peak, IP versus input fold enrichments and *P*-values were calculated.

miRNAs from miRBase (v22.1) were “reverse mapped” to any reads that did not map to repetitive elements or the genome using bowtie (v1.2.3). The miRNA portion of each read was then trimmed, and the remainder of the read was mapped to the genome using STAR (v2.7.7a). PCR duplicates were resolved using umi_tools (v1.1.1), and miRNA target clusters were identified using CLIPper (v2.0.1). Each cluster was annotated with the names of miRNAs responsible for that target. Peaks were annotated using transcript information from GENCODE v41 with the following priority hierarchy to define the final annotation of overlapping features: protein coding transcript (CDS, UTRs, and intron), followed by noncoding transcripts (exon and intron).

### Transfection of miRNA inhibitors and mimics

miRNA mimics were ordered from IDT. The miRCURY LNA^™^ miRNA inhibitors were purchased from QIAGEN. All transfections used Lipofectamine RNAiMAX (Invitrogen). Cells were seeded into six-well plates 24 h before transfection, at 300 K cells per well for wild-type HCT116 cells and 450 K for *DROSHA*-/- cells. At the next day, 50 nM of miRNA mimics or inhibitors were transfected into cells as previously described [50]. Twenty-four hours later, cells were replaced with full culture media. Cells were harvest for quantitative polymerase chain reaction (qPCR) (48 h) after transfection. Full sequence information for miRNA inhibitors and mimics are listed in Supplementary Table S2.

### RT-qPCR

Total RNA was extracted from cells using TRIzol reagent. Reverse transcription was performed using high-capacity reverse transcription kit (Applied Biosystems) as per the manufacturer’s protocol. Two micrograms of total RNA was used per 20 μl of reaction mixture. qPCR was performed on a CFX96 Touch real-time PCR system (Bio-Rad) using iTaq SYBR Green Supermix (Bio-Rad). PCR reactions were done in triplicates at 55°C for 2 min, 95°C for 3 min, 95°C for 30 s, and 60°C for 30 s for 40 cycles in an optical 96-well plate. The primers used are listed in Supplementary Table S1. Data were normalized relative to the level of an internal control gene *RPL19*. Statistical testing was performed using Student’s *t*-test relative to negative control samples.

### RNAPII ChIP-qPCR

Cells were crosslinked with 1% formaldehyde at RT for 10 min and crosslinking was quenched with glycine. Cells were then harvested by scraping in PBS. Cells were pelleted and nuclei were isolated with HLB (10 mM Tris–HCl pH 7.4, 10 mM NaCl, 3 mM MgCl_2_, 2.5% NP-40, and 0.5 mM DTT) and lysed in lysis buffer (50 mM Tris–HCl pH 7.4, 120 mM NaCl, 0.05% NP-40, 1 mM EDTA, and 1 mM DTT). Samples were sonicated for 20 min (30 s on, 30 s off, 50% amplitude, Qsonica Q800R3). Nuclear lysate was precleared, then incubated overnight with RNAPII antibody (05-623, Millipore Sigma, 3 μg) or normal mouse IgG (12-371, Millipore Sigma) in immunoprecipitation buffer. After antibody–protein–DNA complex was recovered with 80 μl of protein G Dynabeads (Invitrogen) for 2 h, the beads were washed with low salt, high salt, LiCl, and TE buffers (1 ml each, 5 min at 4°C). The complex was eluted twice with 250 μl of elution buffer (1% SDS, 0.1 M NaHCO_3_) at RT. Cross-linking was reversed by adding NaCl to a final concentration of 200 mM and heating at 65°C for 2 h. Protein was digested by incubating with proteinase K at 42°C for 50 min, followed by phenol–chloroform extraction and ethanol precipitation. Samples were dissolved in nuclease free water and analyzed by qPCR. Primer sets used are listed in Supplementary Table S1.

### Quantitative polymerase chain reaction (qPCR) splicing assay

A qPCR splicing assay for the *HMGA2* gene was developed by adapting methods as described [51]. A qPCR splicing assay was also developed for the TBP gene as an internal qPCR control. Exon–exon junction spanning primers were designed to have optimal target specificity, melting temperatures, and amplicon size. These primer sets were validated to amplify regions of interest *in silico*. Melt curve analysis for each primer s*et al*so demonstrates robust amplification. Each primer set used in the *HMGA2* and *TBP* qPCR splicing assay is shown in Supplementary Table S1. All primers were synthesized by Integrated DNA Technologies.

Twenty nanograms of complementary DNA (cDNA) corresponding to a given experimental condition was used as a qPCR template for the iTaq^™^ Universal SYBR^®^ Green Supermix reaction (Bio-Rad Laboratories) following the manufacturer’s instructions. All reactions were set-up in 384-well qPCR plates and were run on a CFX384 Touch Real-Time PCR Detection System, followed by preliminary data analysis done in the CFX Maestro Software. As described in Harvey *et al.* (2021), each exon inclusion:skipping ratio was calculated using the formula: 2^−Δ*C*^_t_, where Δ*C*_t_ = (*C*_t_ corresponding to the inclusion mRNA isoform – *C*_t_ corresponding to the skipping mRNA isoform) from the same sample.

Statistical significance in differences between experimental conditions was determined using two-way grouped analysis of variance (ANOVA), and Tukey’s post-hoc multiple comparisons test. All statistical tests were performed in GraphPad Prism 10. Values were determined to be statistically significant if the calculated *P*-value was below an α value of ≤ 0.05.

## Results

### miRNA-associated protein factors in the chromatin fraction

Previous work has detected miRNA-associated proteins and miRNAs in mammalian cell nuclei [9–12, 14, 16, 27, 50] and demonstrated their ability to modulate gene splicing and transcription [13–15, 17–26]. While these data imply that regulation could occur in association with chromatin, direct observation of chromatin-bound AGO or miRNA species has been complicated by competing demands during purification. Isolation of chromatin must be stringent enough to exclude cytoplasm and nucleoplasm but not so stringent as to wash away chromatin-associated AGO protein, AGO:target RNA complexes, and AGO:miRNA complexes. To address these challenges, we chose to optimize conditions for the isolation of cytoplasm and nuclear fractions in HCT116 colorectal cancer-derived cells.

**Figure 1.**
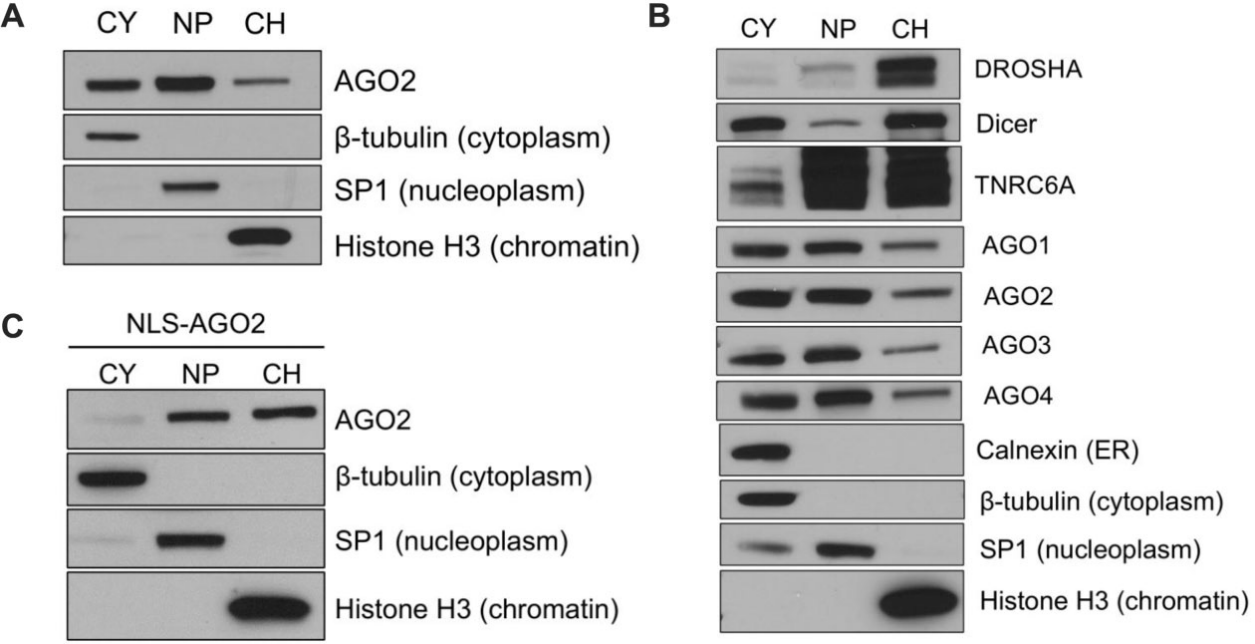
AGO2 and other protein factors involved in miRNA-mediated regulation can associate with chromatin. Western blots showing (**A**) AGO2 localization and purity of cytoplasm (CY), nucleoplasm (NP), and chromatin (CH) in wild-type samples (*N* = 3), (**B**) purity [calnexin for endoplasmic reticulum (ER)] and localization of DROSHA, Dicer, TNRC6A, and AGO1–4 (*N* = 3), and (**C**) AGO2 localization in the nuclear localization sequence (NLS)-AGO2 knock-in cell line (*N* = 3).

We focus on HCT116 cells because we possess CRISPR–Cas9-knockout cell lines lacking critical miRNA-associated protein factors and a CRISPR–Cas9-knockin AGO2-nuclear localization signal line (AGO2-NLS). Starting from published subcellular fractionation protocols [11, 49, 50], we systematically optimized pH, detergent percentage, and salt concentration of our fractionation buffers to identify conditions that prioritized separation of nucleoplasm and chromatin, while maintaining maximum protein content in the chromatin lysate. Following subcellular fractionation, we observed AGO2 protein in the chromatin fraction and confirmed the purity of the fractions using protein markers for the cytoplasm (β-tubulin), nucleoplasm (SP1), endoplasmic reticulum (calnexin), and chromatin (Histone H3) by western analysis (Fig. 1A–C).

There are four AGO protein paralogs in mammalian cells (AGO1–4) with AGO2 being the most abundant in the HCT116 cells used in these studies [52]. AGO2 [4–6], and to a lesser extent AGO3 [53], possess the ability to catalyze the cleavage of RNA substrates. AGO1-4 bind miRNAs similarly [54]. Previous quantitative experiments found that in HCT116 cells, AGO expression follows the trend of AGO2 > AGO1>>AGO2 > AGO4 [52]. Following fractionation into cytoplasm, nucleoplasm and chromatin subcellular fractions, we found that AGO1–4 are present in all compartments of the cells and are associated with the chromatin fraction (Fig. 1B). Because the western analysis uses different anti-AGO antibodies to detect each AGO variant, the relative amounts of the AGO variants cannot be determined from the western analysis.

Drosha and Dicer are the RNase III enzymes which catalyze the production of mature miRNAs and function in the nucleus and cytoplasm, respectively. We found that Drosha is mainly present in chromatin, while Dicer is present in the cytoplasm and chromatin (Fig. 1B), consistent with previous results [11, 55, 56].

Human GW182 or trinucleotide repeat containing protein 6A (TNRC6A) is a scaffolding protein that interacts with AGO2 and facilitates miRNA-mediated degradation of transcripts [57–63]. This scaffolding function contributes to cooperative binding of multiple AGO complexes and bridges interactions with other functional proteins.

Our data show that human TNRC6A is present in all compartments of the cell, including chromatin (Fig. 1B), suggesting that it is available to act as a scaffold to promote complex formation with other proteins. We note that our gel loading involves equal protein, not equal cell equivalents. Our analysis is designed to evaluate purity and does not represent the absolute levels of TNRC6A or other proteins in the different chromatin, nucleoplasm, or cytoplasm relative to one another. We have not attempted to optimize recovery of cytoplasmic proteins since our focus is on chromatin and our results do not imply that there is more TNRC6A in cell nuclei relative to cytoplasm. We and others have reported previously nuclear TNRC6A [11, 16, 35, 60, 61] and used mass spectrometry to characterize its interacting protein partners in nuclear lysate [16, 57]. Similar uncertainty due to protocols being optimized for purity rather than quantitative recovery applies to the relative nuclear localization of Dicer and the AGO variants.

We used our NLS–AGO2 HCT116 cells to provide us with an alternative method for examining the nuclear interactions of AGO2 by chimeric eCLIP that is independent of subcellular fractionation. This NLS–AGO2 cell line has a nuclear localization sequence added to the N-terminus of the endogenous locus of the AGO2 gene. This construct avoids overexpression, but forces endogenous AGO2 to localize to the nucleus [50] and chromatin (Fig. 1C). Therefore, immunoprecipitation using an anti-AGO2 antibody will isolate nuclear AGO2:RNA interactions without the need for a prior subcellular fractionation step, providing a complementary strategy for examining nuclear associations.

### Scheme for chimeric eCLIP

Interactions between miRNAs and cellular RNA targets can be predicted by computation. However, it can be difficult to sort through the many different predicted targets. To increase the likelihood that time-consuming experimental validation is focused on the most promising targets, it is useful to complement computational analysis with physical methods that detect sites where physical interactions between miRNAs, AGO proteins, and RNA targets occur.

**Figure 2.**
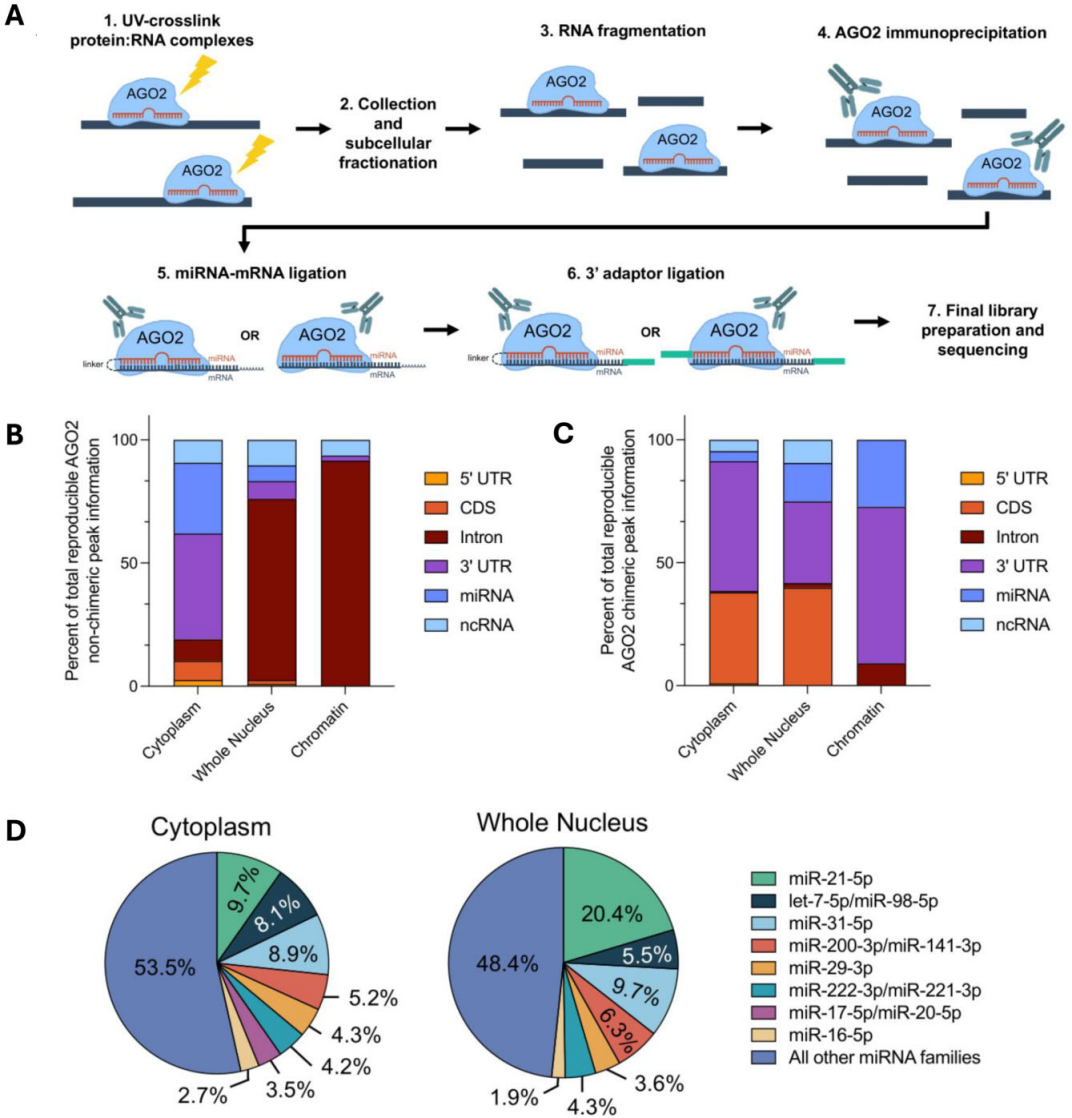
AGO2 interacts with target RNAs in all cellular compartments. (**A**) Schematic outlining the chimeric eCLIP protocol used for the identification of AGO2-binding sites. Distribution of reproducible AGO2 (**B**) nonchimeric and (**C**) chimeric binding peaks across genomic features in cytoplasm (*N* = 2), whole nucleus (*N* = 2), and chromatin (*N* = 2) fractions. Chimeric binding peaks only include chimeric reads that include miRNAs that map to the miRGeneDB database [40]. (**D**) Percentage of chimeric miRNAs from the top eight miRNA families sharing the same seed sequence.

We chose to use AGO2 chimeric enhanced crosslinking and immunoprecipitation (chimeric eCLIP) to identify these physical interactions between miRISC and its RNA targets [48] (Fig. 2A). eCLIP employs UV crosslinking prior to harvesting cells, allowing for detection of interactions that occur within intact, living cells. Cellular fractions are then obtained and an anti-AGO2 antibody is used to pull down the protein:RNA complex. To increase the likelihood of detecting direct miRNA–RNA target interactions, the immunoprecipitation is followed by a ligation step that connects a miRNA to its mRNA target.

The ligation step that joins miRNA and RNA target is not efficient and yields two groups of RNA products following digestion of the AGO2 protein. miRNA–mRNA pairs where the ligation was successful will be chimeric, with a linker connecting them. These chimeric reads identify direct miRNA binding to targets—information that establishes interactions with high confidence. The remaining reads, a much larger portion, will remain nonchimeric and identify RNAs that associate with AGO2 (Fig. 2A).

Both nonchimeric and chimeric reads are derived from the same sample, representing parts of the whole regarding AGO2 binding. Chimeric reads identify direct miRNA:target interactions while nonchimeric reads identify AGO2 bound RNAs. Nonchimeric binding events are inferred to be mediated by a miRNA, but the specific miRNA must be predicted based primarily on sequence information. A miRNA:target interaction that is determined from nonchimeric reads is less certain evidence for miRNA:RNA recognition. There are, however, more nonchimeric reads, providing a deeper pool of candidate gene targets. Together, chimeric and nonchimeric offer a wide pool of insight into AGO2 binding that can be used for prioritization of genes for experimental validation.

To gain insight into whether functional targets for miRNAs could be identified in chromatin, we performed anti-AGO2 chimeric eCLIP experiments in duplicate in four different sample types. These included whole cell lysate, isolated cytoplasm lysate, and isolated chromatin lysate from wild-type HCT116 cells. We also evaluated whole cell lysate from NLS–AGO2 cells to observe whole nucleus AGO2 interactions without the need for subcellular fractionation (Supplementary Table S3 and Supplementary Fig. S1).

### Chimeric eCLIP:RNA target regions and miRNAs

Both total (∼56–62 million) and final nonchimeric (9.5–16.6 million) reads were similar in every sample regardless of whether the original was whole cell, cytoplasm, whole nucleus, or chromatin (Supplementary Table S3). In all samples, the number of chimeric reads was much lower than the number of nonchimeric reads because the generation of chimeric reads involves a ligation step that limits the efficiency of detection of the ligation step that allows generation of chimeric reads. Lower detection of chimeric reads is consistent with prior results using other methods that report ligation efficiencies ranging from 1.5% to 5% [46]. Chimeric reads were at least 10-fold lower in the chromatin sample (∼14 000 reads) relative to whole cell (∼100 000 reads), cytoplasm (∼170 000 reads), and nuclear (75 000 reads) samples.

For cytoplasmic RNA, most nonchimeric reads were associated with recognition within the 3′-untranslated region (3′-UTR) (Fig. 2B and Supplementary Fig. S2A). By contrast in the nucleus and chromatin fraction, most peaks are observed within intronic RNA, likely because of the much greater fraction of intronic RNA present prior to splicing and nuclear export. Chimeric AGO2 peaks in the cytoplasm were mainly found within the 3′-UTR (Fig. 2C and Supplementary Fig. S2B), like the nonchimeric binding peaks. By contrast to the similar results in cytoplasm for nonchimeric and chimeric AGO2 peaks, data from nuclei and chromatin showed a shift to binding within the 3′-UTR (Fig. 2C and Supplementary Fig. S2B).

Why would chimeric reads in the chromatin or nuclear fractions cluster within 3′-UTRs whereas nonchimeric reads cluster within introns? We hypothesize that the greater percentage of chimeric reads within the 3′-UTR in nucleus or chromatin (Fig. 2C and Supplementary Fig. S2B) is due to miRNA:AGO2:3′-UTR complexes being stronger and hence more detectable by the relatively less efficient chimeric eCLIP. In addition, many intronic RNAs will likely be present at lower concentrations than well expressed mature mRNAs like *HMGA2*.

The chimeric reads also contain information about the specific miRNAs mediating target RNA recognition (Fig. 2D). Using the reads for the top 20 miRNAs in the chimeric reads from whole cell wild-type HCT116 eCLIP samples, we found that eight miRNA families make up ∼50% of the total reads. In the cytoplasm and nucleus, these eight miRNA families account for 46.5% and 51.6% of the chimeric miRNA reads (Fig. 2D). The number of chimeric peaks in the chromatin fraction was too small to draw conclusions about the broader distribution of miRNAs (but was adequate to identify individual targets, see below).

### Prioritizing genes for experimental analysis

To facilitate experimental validation, we prioritized genes that (i) had >5 chimeric AGO2 peaks in the 3′-UTR in either the cytoplasm, whole nucleus, or chromatin or at least one chimeric peak in all samples (cytoplasm, nucleus, and chromatin) and (ii) had altered expression in wild-type versus *DROSHA-/-* cells. Drosha protein is the most upstream enzyme in the miRNA biogenesis pathway. Knockout of the protein leads to depletion of most mature miRNAs [54] and gene expression changes would suggest a potential miRNA-mediated effect on gene expression. From this prioritization, we chose five genes for experimental analysis, *HMGA2*, *MYC*, *ZFP36*, *ZP36L1*, and *TNFRSF12A* (Supplementary Table S4). All these genes have at least one chimeric peak in their 3′-UTR in the cytoplasm and nucleus and *HMGA2, MYC*, and *TNFRSF12A* have chimeric peaks detected in chromatin. Each gene also has nonchimeric AGO2 peaks within their 3′-UTRs of varying strengths (Supplementary Fig. S3). The *DROSHA*-/- cells were used for comparative analysis relative to wild-type HCT116 cells to evaluate the effects of removing most miRNAs from HCT116 cells.

**Figure 3.**
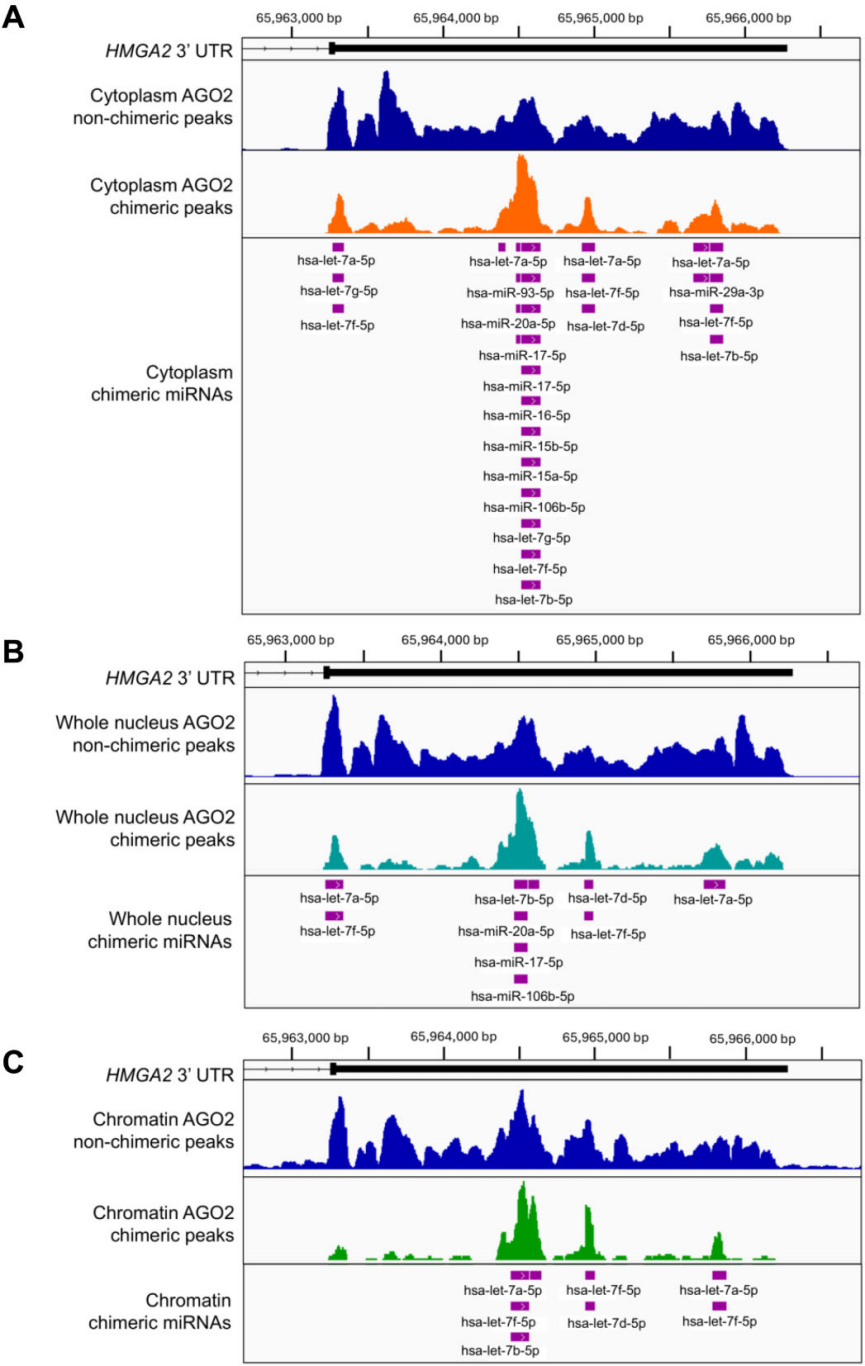
*HMGA2* RNA is bound by AGO2 in all compartments of the cell. Representative IGV browser images of chimeric eCLIP reads within the 3′-UTR of *HMGA2* in the (**A**) cytoplasm, (**B**) nucleus, and (**C**) chromatin. *HMGA2* is located on chromosome 12 (chr12). Peak height is defined as read density in reads per million (RPM). All AGO2 peaks are reproducible and meet the following criteria: log2FC > 3 (IP versus input) at least three reads per peak.

**Figure 4.**
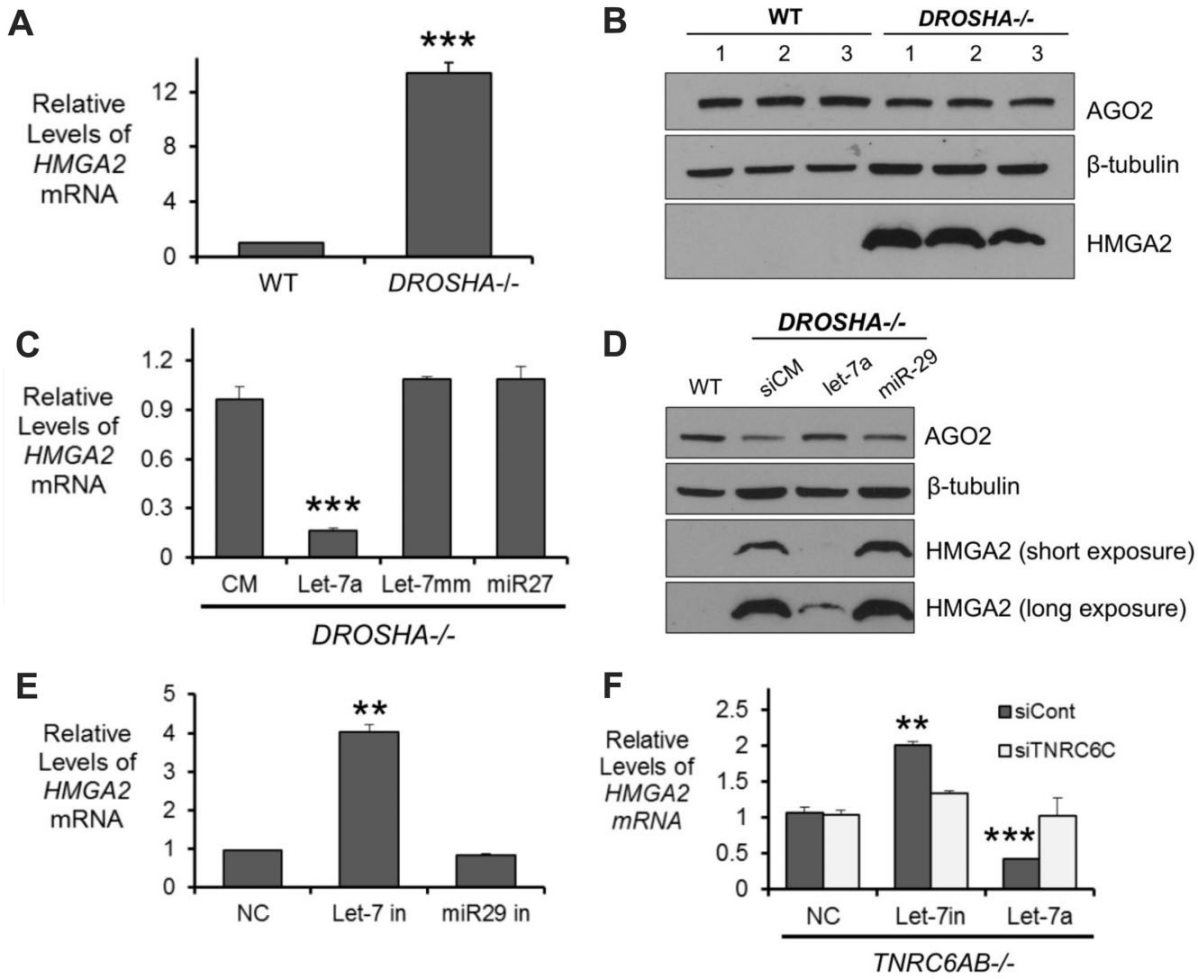
*HMGA2* expression is regulated by the *let-7* family of miRNAs. (**A**) Relative expression of mature *HMGA2* mRNA in WT HCT116 and *DROSHA-/-* cells. (**B**) Western blot validation of HMGA2 protein expression in three biological replicates of untreated WT HCT116 and DROSHA-/- (HMGA2, 3-min exposure). (**C**) Relative expression of mature *HMGA2* mRNA in *DROSHA-/-* cells transfected with control (siCM, let-7mm, and miR-27) and let-7a mimics. (**D**) Western blot validation of HMGA2 protein expression in DROSHA-/- cells treated with control and let-7a miRNA mimics. HMGA2 short exposure (30 s) and long exposure (10 min). (**E**) Relative expression of mature *HMGA2* mRNA in WT HCT116 transfected with negative control oligonucleotides (NC and miR-29 family inhibitor) or let-7 family miRNA inhibitors. (**F**) Relative expression of *HMGA2* mature mRNA in TNRC6AB-/- HCT116 cells were transfected with control, let-7 family miRNA inhibitor and let-7a miRNA mimic. All values are plotted as the average of biological replicates ± SD. Significance denoted as **P< 0.01, ***P< 0.001.

Of these gene candidates, *high mobility group AT-Hook 2* (*HMGA2)* was the most compelling. Both nonchimeric and chimeric AGO2 peaks were detected in the 3′-UTR of *HMGA2* in the cytoplasm, nucleus and chromatin (Fig. 3, and Supplementary Figs S3–S5 and Supplementary Table S4), and HMGA2 expression in *DROSHA*-/- cells was upregulated relative to wild-type (Fig. 4A). Together, these data suggest a potential de-repression of miRNA-mediated silencing and is consistent with canonical gene silencing mechanisms.

### HMGA2, a model for miRNA-mediated recognition

miRNA-mediated gene silencing is most effective when multiple miRNAs bind at adjacent sites [64–66]. Binding of multiple miRNAs in proximity to one another permits cooperative interactions through the scaffolding protein TNRC6 which can bind up to three AGO proteins at one time. This allows TNRC6 to bridge three AGO:miRNA complexes and stabilize their binding to target mRNA [62].

For example, within the 3′-UTR of *HMGA2*, chimeric eCLIP detected >20 miRNA binding peaks in the cytoplasm (Fig. 3A and Supplementary Fig. S4) and 8 miRNA binding peaks in the nucleus and chromatin (Fig. 3B and C, and Supplementary Fig. S5). Of these binding peaks, we observed four binding sites for the *let-7* family of miRNAs using whole cell, nuclear or cytoplasmic anti-AGO2 chimeric eCLIP (Fig. 3A and B, and Supplementary Figs S4 and S5A and B), and three binding sites by chimeric eCLIP of chromatin (Fig. 3C and Supplementary Fig. S5C and D). Comparative analysis suggests that the *let-7* seed sequence at these binding sites are well-conserved (Supplementary Fig. S6).

The involvement of *let-7* is consistent with previous computational and experimental analyses implicating *let-7* in the regulation of *HMGA2* expression in other cancer-derived cell lines [67–70]. The *let-7* family is one of the more prevalent miRNAs detected by chimeric eCLIP (Fig. 2D). The combination of compelling chimeric eCLIP data and data implicating *let-7* in the control of HMGA2 in other cell types led us to focus on exploring the *let-7/HMGA2* axis as a model for understanding the contributions of nuclear and cytoplasmic interactions to gene silencing.

### HMGA2, a model for miRNA-mediated gene regulation

To find experimental support for the role of *let-7* family miRNAs in the regulation of *HMGA2* gene expression in HCT116 cells, we used complementary methods for probing miRNA-mediated regulation using RNA isolated from HCT116 whole cell lysate. As mentioned, Drosha is the protein responsible for production of most mature miRNAs and its knockout leads to the depletion of most mature miRNAs in HCT116 cells [54]. Measurement of *HMGA2* levels by qPCR showed that, relative to WT HCT116, the *HMGA2* transcript is upregulated, >10-fold in *DROSHA*-/- cells (Fig. 4A).

Western analysis of protein levels revealed no detectable expression of HMGA2 protein in wild-type HCT116 cells, even after long exposure of film after blotting (Fig. 4B). Expression of HMGA2 protein was increased in *DROSHA*-/- HCT116 cells. This result is impressive evidence of the power of miRNA-mediated silencing to control protein expression even more stringently than RNA expression.

While analysis of the *DROSHA*-/- cells confirmed that miRNAs are involved in *HMGA2* regulation, we next wanted to determine whether the *let-7* family of miRNAs that we had identified through eCLIP play a direct role in this regulation. Duplex RNAs that mimic the sequence of *let-7* would be expected to also mimic the ability of *let-7* to repress gene expression [71]. To test this hypothesis, we used *DROSHA*-/- HCT116 cells that lack endogenous expression of *let-7* miRNAs [54]. Following transfection of a *let-7a* mimic, *HMGA2* expression was decreased relative to a noncomplementary control duplex, a *miR-27* mimic, or a mismatched duplex based on the *let-7a* sequence (Fig. 4C). We also observed that HMGA2 protein expression follows the same trend (Fig. 4D). The ability of *let-7a* mimics to suppress *HMGA2* expression suggests that *let-7* family miRNAs play a dominant role in regulation even though there are predicted binding sites for other miRNAs.

Synthetic oligonucleotides that are complementary to miRNAs (miRNA inhibitors or antimiRs) can block the action of miRNAs and reverse their control of gene expression [72–74]. To test the effect of *let-7* miRNAs on *HMGA2* expression, we used a *let-7* miRNA family inhibitor that selectively blocks activity of the *let-7* family members in wild-type HCT116 cells. Transfection of the *let-7* family miRNA inhibitor increased *HMGA2* expression (Fig. 4E), while a miRNA inhibitor targeting the *miR-29* family or a noncomplementary control had no effect on *HMGA2* expression (Fig. 4E). Together, these results further support that the *let-7* family of miRNAs play a direct role in the regulation of *HMGA2* expression.

TNRC6 is a scaffolding protein that interacts with AGO2 and is critical for miRNA-mediated inhibition of translation [75–80]. AGO2 also retains catalytic activity that does not require TNRC6 when the guide RNA and target RNA are perfectly complementary [4–6, 52]. To separate these two potential pathways of miRNA-mediated silencing, we chose to use our *TNRC6AB-/-* cells to examine the necessity of the TNRC6 paralogs for HMGA2 silencing. TNRC6 has 3 paralogs, and in the *TNRC6AB-/-* cell line, TNRC6C protein expression is upregulated [52]. As a result, testing the effects of reducing expression of *TNRC6A*, *TNRCB* and *TNRC6C* is best accomplished by introducing an siRNA designed to reduce TNRC6C into TNRC6AB-/- cells.

When we transfected miRNA inhibitor targeting the *let-7* family into *TNRC6AB-/-* cells (elevated *TNRC6C* expression, not a *TNRC6* family knockout), we observed increased *HMGA2* expression, consistent with residual *let-7* mediated gene silencing (Fig. 4F). When *TNRC6C* was also silenced using an siRNA, *HMGA2* expression returned to control level, consistent with the TNRC6 paralogs acting together to contribute to miRNA-mediated regulation of *HMGA2*. These data are consistent with previous work suggesting that the TNRC6 paralogs can compensate for one another and loss of all three is necessary to observe their full impact [26, 52].

When a *let-7a* mimic was transfected into *TNRC6AB-/-* cells, we observed a decrease in HMGA2 expression, consistent enhanced silencing by the synthetic miRNA. When *TNRC6C* was also silenced, addition of the *let-7a* mimic had no significant effect on gene expression (Fig. 4F). While not definitive with regards to mechanism, our data modulating *let-7* activity in *TNRC6* deficient cells is consistent with the conclusion that *HMGA2* silencing may occur through a mechanism dependent on the TNRC6 family of proteins, like the canonical miRNA-mediated mechanism of translational repression.

### Regulation of other candidate genes

We also examined the potential for miRNA-mediated regulation of our other candidate genes, *MYC*, *ZFP36*, *ZFP36L1*, and *TNFRSF12A*. For *TNFRSF12A*, we observed nonchimeric and chimeric AGO2 binding in the 3′-UTR and binding by miR-19 in the cytoplasm, whole nucleus, and chromatin (Supplementary Figs S7A–C and S8), and these binding sites are evolutionarily well conserved (Supplementary Fig. S9). Transfection of miR-19a miRNA mimics in a *DROSHA*-/- background led to silencing of the *TNFRSF12A* mature transcript in whole cell, cytoplasm and nucleus (Supplementary Fig. S7D and F), but had no impact on the pre-mRNA (Supplementary Fig. S7E and G). While not as in depth as *HMGA2*, *TNFRSF12A* is an additional example of a gene candidate identified through chimeric eCLIP that seems to be regulated by miRNAs.

For *MYC*, we observed nonchimeric AGO2 association and chimeric *let-7* reads within the *MYC* 3′-UTR (Supplementary Figs S10A and S11). The *let-7* seed sequence binding sites were relatively well conserved (Supplementary Fig. S12). However, despite this evidence for AGO2 association, *MYC* expression was decreased in *DROSHA*-/- cells—a result opposite to what would be expected by canonical miRNA-mediated regulation (Supplementary Fig. S10B and Supplementary Table S4).

We observed no direct *let-7* mediated impact on *MYC* gene expression. In *DROSHA-/-* cells, the transfection of *let-7a* mimics had no significant impact on the expression of *MYC* mRNA (Supplementary Fig. S10C). In wild-type cells, addition of *let-7* family miRNA inhibitors had no significant effect on *MYC* expression in cell cytoplasm or nuclei (Supplementary Fig. S10D and E). We observed a similar lack of correlation between AGO2 chimeric and nonchimeric binding and regulation of gene expression at the paralog *ZFP36* and *ZFP36L1* loci (Supplementary Figs S13–S16). Taken together, these data indicate that the presence of chimeric reads within a gene is no guarantee of observable biological regulation by candidate miRNAs.

### let-7 regulates HMGA2 RNA levels in cell nuclei

After establishing an experimental foundation to support *let-7* mediated regulation of *HMGA2* expression in whole cell lysate (Figs 3 and 4), we investigated whether the observed strong recognition of the *HMGA2* 3′-UTR by *let-7* within the nucleus and chromatin (Fig. 3B and C) contributes to the regulation of *HMGA2* silencing. To test this hypothesis, we performed subcellular fractionation and evaluated nuclear and cytoplasmic levels of *HMGA2* in *DROSHA*-/- cells. We also tested the impact of miRNA inhibitors and miRNA mimics on HMGA2 mRNA expression in the cytoplasm and nucleus. For comparison, we fractionated untreated WT and *DROSHA-/-* HCT116 cells and evaluated *HMGA2* expression in both the nuclear and cytoplasmic fractions.

**Figure 5.**
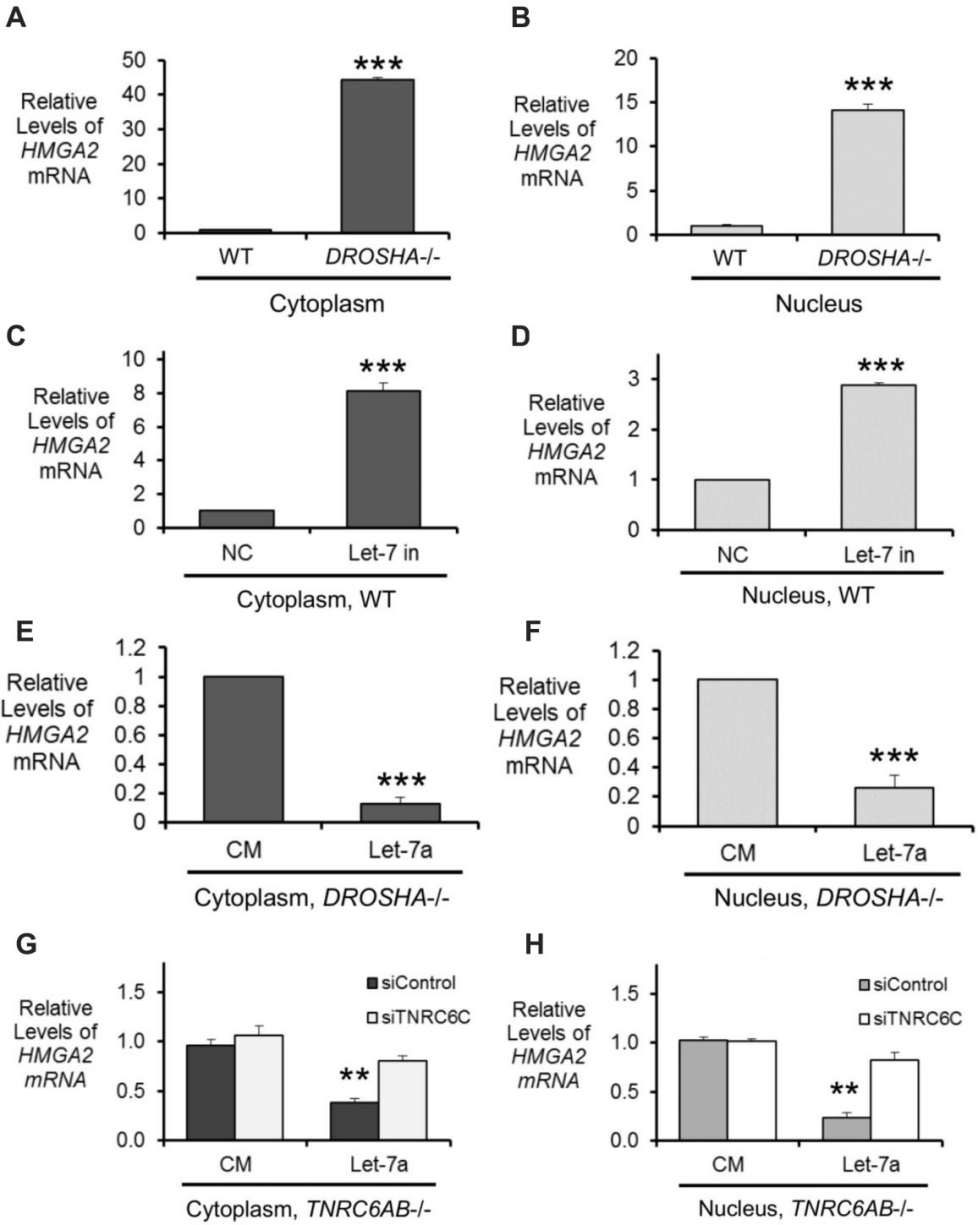
miRNA-mediated repression of HMGA2 can occur in the cytoplasm and nucleus. Relative level of mature *HMGA2* mRNA in untreated WT HCT116 and DROSHA-/- (**A**) cytoplasm and (**B**) nucleus. Relative level of mature mRNA transcript in the (**C**) cytoplasm and (**D**) nucleus of HCT116 WT transfected with control (NC) or *let-7* family miRNA inhibitor. Relative level of mature HMGA2 transcript in the (**E**) cytoplasm and (**F**) nucleus of DROSHA-/- transfected with control (CM) and let-7a mimic. Relative level of mature *HMGA2* transcript in the (**G**) cytoplasm and (**H**) nucleus of *TNRC6AB-/-* cells with knockdown of *TNRC6C*, followed by transfection of *let-7a* miRNA mimic or control (CM) double stranded RNAs. All values are plotted as the average of biological replicates ± SD. Significance denoted as **P< 0.01, and ***P< 0.001.

First, we examined the impact on *HMGA2* expression in the cytoplasm, the canonical site of miRNA-mediated silencing. We observed that *HMGA2* expression was increased in the cytoplasm of *DROSHA*-/- cells relative to wild-type (Fig. 5A). Inhibition of the *let-7* family of miRNAs led to an increase in HMGA2 expression (Fig. 5C), and addition of a *let-7a* miRNA mimic reduced expression of *HMGA2* in *DROSHA*-/- cells (Fig. 5E). As in whole cell, the silencing of *HMGA2* in the cytoplasm was dependent on the presence of the TNRC6 paralogs (Fig. 5G). These results suggest that in the cytoplasm, *HMGA2* is silenced through miRNA-mediated recognition of its 3′-UTR by *let-7* miRNA loaded miRISC and subsequent translational repression, consistent with the canonical mechanism for cytoplasmic gene silencing by miRNAs.

Our examination of the nuclear fraction revealed similar miRNA-mediated regulation of gene expression. In the nuclear fraction of *DROSHA*-/- the expression of *HMGA2* was increased relative to WT (Fig. 5B). Transfection of *let-7* family miRNA inhibitors led to an increase in HMGA2 expression (Fig. 5D) while transfection of a *let-7a* miRNA mimic in *DROSHA*-/- cells led to a decrease in expression (Fig. 5F). Further, silencing of HMGA2 in the nucleus was dependent on the TNRC6 paralogs, like in the cytoplasm (Fig. 5H). These observations support the hypothesis that binding of AGO2:miRNA complexes within chromatin-associated RNA and nuclear RNA may also contribute to the silencing of *HMGA2*.

To determine whether *let-7* mediated inhibition of HMGA2 expression in cell nuclei is observed in other cells, we evaluated HT29 and HeLa cells. The expression of HMGA2 in HT29 and HeLa cells is lower than in HCT116 cells (Supplementary Fig. S17A). Despite the difference in expression levels, *let-7* mediated regulation was similar. Addition of *let-7* family miRNA inhibitors led to an increase in HMGA2 expression in whole cell (Supplementary Fig. S17B and D), cytoplasm, and nucleus (Supplementary Fig. S17C and E).

### HMGA2 expression is not controlled at the level of transcription or splicing

Previous studies from our laboratory and others had demonstrated that synthetic duplex RNAs can control gene transcription and gene splicing [13–28]. These previous regulatory duplex RNAs, however, did not target the 3′-UTR. For regulating transcription, the duplex RNAs were complementary to noncoding RNAs that overlap the 5′ or 3′ terminus of target mRNAs [14, 81–83]. For gene splicing, they were targeted near intron/exon junctions and splicing regulatory elements [19–21]. While the regulatory region within *HMGA2* 3′-UTR is different from these previous studies, the precedent for regulating transcription or splicing led us to explore the potential for changes in *HMGA2* transcription and splicing.

**Figure 6.**
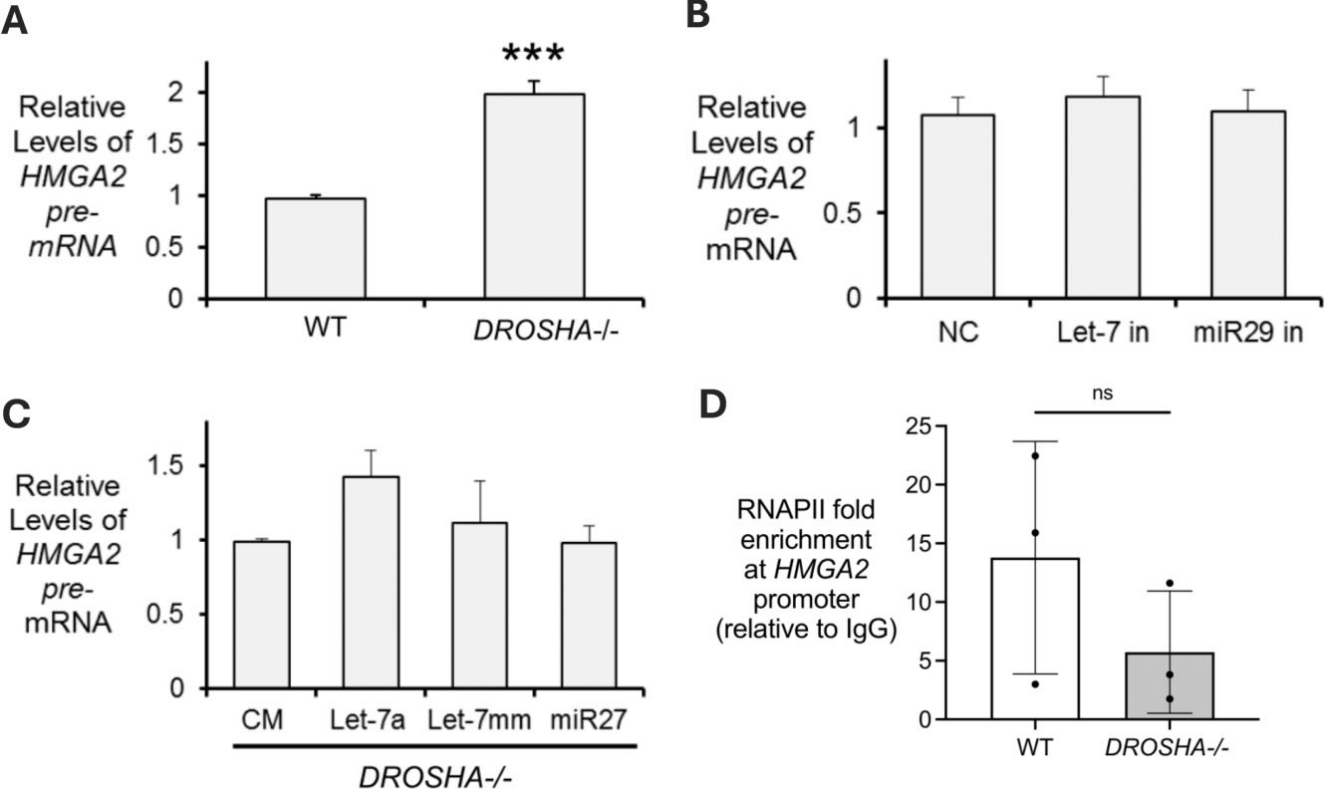
*HMGA2* expression is not transcriptionally regulated by *let-7* family miRNAs. Relative *HMGA2* pre-mRNA levels in (**A**) wild-type and *DROSHA-/-* cells, (**B**) wild-type cells treated with nontargeting oligonucleotide control (NC), *let-7* family or *miR-29* family miRNA inhibitor, and (**C**) *DROSHA-/-* cells transfected with *let-7a* or control double-stranded miRNA mimics (CM, miR-27, let-7mm). (**D**) ChIP-qPCR measuring the recruitment of RNA polymerase II to the *HMGA2* promoter in WT and *DROSHA-/-* cells. All values are plotted as the average of biological replicates ± SD. Significance denoted as ***P< 0.001.

To test the hypothesis that *let-7* miRNAs may regulate the *HMGA2* transcript through modulation of transcription, we first examined the pre-mRNA transcript expression levels. In *DROSHA-/-* cells, we observed an increase in the *HMGA2* pre-mRNA level (Fig. 6A and Supplementary Fig. S18A and B). While these data are consistent with a miRNA-mediated effect of *HMGA2* transcription, they do not indicate that the effect is specific to an interaction at the *HMGA2* gene.

To evaluate the potential for a specific *let-7*-mediated control of HMGA2 expression, we used let-7 family miRNA inhibitors or *let-7a* mimics. Following transfection of *let-7* miRNA family inhibitors in WT (Fig. 6B, and Supplementary Fig. S18C and D) or *let-7a* miRNA mimics in *DROSHA-/-* cells (Fig. 6C and Supplementary Fig. S18E and F), we observed no significant change in the level of *HMGA2* pre-mRNA.

Finally, we performed RNAPII ChIP-qPCR to measure the recruitment of RNA polymerase II to the *HMGA2* promoter. Relative to WT, we observed no change in the recruitment of RNAPII to the *HMGA2* promoter in the *DROSHA*-/- cells (Fig. 6D and Supplementary Fig. S19). Taken together, these data are consistent with the conclusion that indirect effects of miRNAs may increase *HMGA2* transcription, but do not suggest a significant impact from *let-7* binding to chromatin-associated RNA.

**Figure 7.**
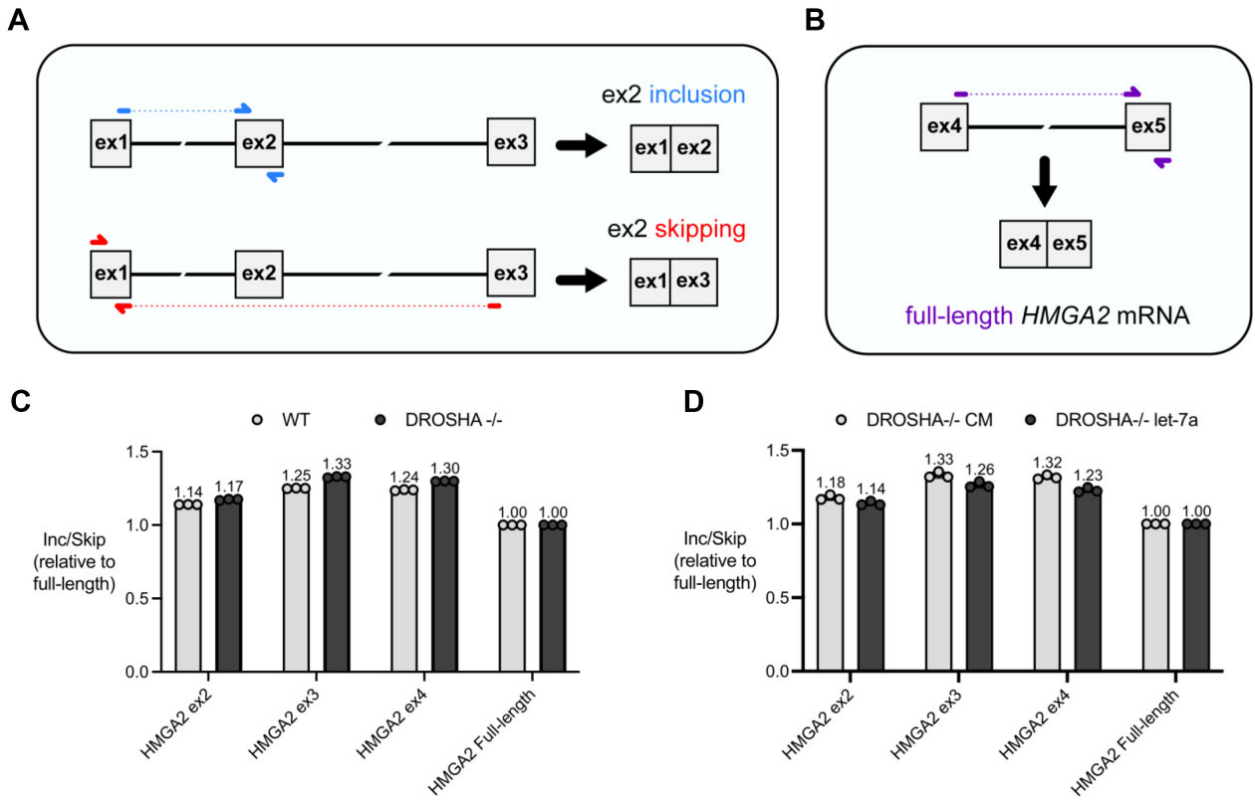
pre-mRNA splicing of *HMGA2* is not regulated by *let-7* family miRNAs. (**A**) A representative schematic using *HMGA2* exon 2 to depict the exon-exon junction spanning primers designed for the measurement of exon inclusion and/or skipping events within the *HMGA2*gene. The presence of solid-colored lines indicate direct complementarity of primers to its target sequence, whereas dashed-colored lines indicate no complementarity. (**B**) A schematic depicting an exon-exon junction spanning primer set designed to measure the total abundance of full-length *HMGA2* transcripts. The results from this assay are plotted as splicing inclusion:skipping ratios, as shown in (**C**) for untreated WT and *DROSHA*-/- cells and (**D**) for *DROSHA*-/- transfected with *let-7a* miRNA mimic or control duplex (CM). Each condition's respective mean value, calculated from three independent biological replicates, are displayed above each condition shown.

Alternatively, a change in *HMGA2* expression could also be driven by changes in pre-mRNA splicing. To test this hypothesis, we designed a locus-wide qPCR splicing assay to measure how often a given *HMGA2* exon is included in spliced mRNA compared to how often it is skipped (Fig. 7A). Primers designed to span exon-exon junctions allow for the specific detection of mRNA isoforms that include or skip a given *HMGA2* exon (Supplementary Table S1). Each exon’s inclusion:skipping ratio, normalized to the expression of the full-length mRNA, is therefore representative of an exon’s splicing efficiency.

We first applied this assay to understand how loss of mature miRNAs in the *DROSHA*-/- cells might affect *HMGA2* gene splicing. Relative to WT HCT116 cells, we observed that the splicing efficiency of each *HMGA2* exon is similar in the DROSHA-/- and wild-type cells (Fig. 7B). To further test the hypothesis that *let-7* miRNAs may regulate *HMGA2* gene splicing, we repeated the same splicing assay using *DROSHA*-/- cells transfected with either a *let-7a* mimic or a noncomplementary (CM) control. We observed only minor differences in splicing efficiency relative to the CM control (Fig. 7C). A similar qPCR splicing assay done in parallel targeting the reference gene *TBP*, an internal control for each experimental condition presented, demonstrates that miRNA-mediated splicing modulation of *HMGA2* by *let-7a* is sequence specific (Supplementary Fig. S20). Our data do not support the conclusion that *let-7* binding has a major effect on splicing within *HMGA2* mRNA.

## Discussion

### Cytoplasmic and nuclear miRNA-mediated regulation of gene expression

Complexes between AGO and RNA are programmable recognition elements. AGO protein protects the RNA (either endogenous miRNA or synthetic designed duplex RNA) and facilitates target recognition. The small RNA component directs the complex to target sequences. While the canonical targets for miRNAs are within the 3′-UTRs of cytoplasmic RNAs [1–3], there is no theoretical reason why sites outside the 3′-UTR cannot be targeted, including targets within mammalian cell nuclei.

Synthetic duplex RNAs can complex with AGO to control gene transcription [13–18] and regulate gene splicing [19–21]. AGO:RNA complexes complementary to the disease-associated repeat RNAs of Friedreich’s Ataxia [84, 85], Fuchs Dystrophy [86, 87], and C9orf72-related ALS [88] can block R-loop formation or protein association. Localization of AGO protein to cell nuclei when cells are grown to high confluence can de-repress cytoplasmic miRNA targets [50].

While these reports are intriguing and suggest versatile mechanisms for nuclear miRNAs and their regulation, most work has focused on the action of synthetic, designed RNAs. The unanswered question is whether the robust impacts of designed duplex RNAs in cell nuclei are reflected in widespread, biologically significant effects on the regulation of cellular gene expression by miRNAs. Our results have identified the potential for miRNAs to bind chromatin-associated RNA and silence endogenous nuclear targets.

Previous work from our laboratory [11, 57] and from Sarshad *et al.* [16] revealed that protein factors involved in the miRNA pathway were present and formed complexes in cell nuclei. Here, we confirm that chimeric eCLIP is a robust methodology for understanding the details of miRNA-mediated gene regulation, that AGO2:miRNA complexes assemble on chromatin, and that canonical miRNA-mediated gene silencing in the cytoplasm may be supplemented by gene silencing in cell nuclei. As chimeric eCLIP and other analytical techniques become more powerful, and as other cellular systems are investigated, it is possible that a broader role for biological regulation by nuclear miRNAs might become apparent. These potential roles for nuclear miRNAs may reveal new insights into endogenous processes and new starting points for the design of therapeutic agents that target RNA.

### Identification HMGA2:let-7 regulation using anti-AGO2 chimeric eCLIP

Identifying suspected interactions through seed sequence complementarity between miRNAs and RNA targets can be accomplished with computational analysis. Computational analysis, however, will identify many potential sites for biological regulation. The challenge is to identify methods that prioritize sites that are most likely to be significant for cellular regulation to justify the experimental effort necessary for rigorous validation.

To meet our criteria as a “high priority target,” a site was required to meet the following criteria: (i) nonchimeric AGO2 peak density, (ii) at least five chimeric AGO2 peaks in one fraction or at least one chimeric AGO2 peak in all fractions, (iii) the miRNAs revealed by chimeric eCLIP belong to well-expressed miRNA families, and (iv) a significant change in the expression of target genes in wild type versus *DROSHA*-/- cells.

Our chimeric eCLIP data revealed few genes that met these criteria (Supplementary Table S4). Further experimental validation showed that one of these candidates (Fig. 3–5 and Supplementary Figs S4–S7), *HMGA2*, was a strong candidate for direct repression by miRNAs in HCT116 cells. Regulation by *let-*7 family miRNAs was supported by data showing rescued gene silencing in *DROSHA-/-* cells following *let-7a* miRNA mimic transfection (Fig. 4C and D), increased expression upon addition of *let-7* family miRNA inhibitors (Fig. 4E), and modulation in *HMGA2* expression by inhibitors and mimics following knockout and knockdown of the TNRC6 paralogs (Fig. 4F).

While *let-7* family miRNAs have been characterized as a regulator of *HMGA2* in other cell types [67–70], it is a good example of the power of chimeric eCLIP for prioritizing candidates. A PubMed search of “miRNA” and HCT116 reveals over 1300 publications. Chimeric eCLIP identified *HMGA2* as a priority candidate for experimental validation in HCT116 cells, even though many reports implicated other miRNAs and other gene targets. Several of these publications implicate *HMGA2* as a target, but to our knowledge none discuss *let-7* as partner. Other publications discuss *let-7*, but do not focus on *HMGA2*.

In contrast to the diversity of results found in the literature, chimeric eCLIP unambiguously identified nonchimeric AGO2 association and chimeric binding peaks in the *HMGA2* 3′-UTR in multiple subcellular fractions. These chimeric binding interactions were predominately found to involve *let-7* family miRNAs, one of the most abundant miRNA families in HCT116 cells. Our experimental data (Figs 4 and 5) validated the priority assigned to *HMGA2* by the chimeric eCLIP data.

### Nuclear recognition and regulation

Our replicate chimeric eCLIP data using chromatin from wild-type HCT116 cells reveals binding of AGO2 and chromatin-associated RNA throughout the transcriptome. While most nonchimeric reads are within introns, most chimeric reads and the most promising candidate interactions are within 3′-UTRs. The distribution of chimeric reads to the 3′-UTR, even in our nuclear datasets, likely reflects the strength of binding of AGO2:miRNA complexes to target 3′-UTRs. Our data from HCT116 AGO2–NLS cells where AGO2 is almost exclusively nuclear show similar distribution of binding for miRNA:AGO2 complexes. Our replicate data from HCT116 cytoplasm is also similar.

Our data identified *HMGA2* and a handful of other genes as prime candidates for miRNA-mediated recognition of chromatin-associated RNA. Experimental validation revealed that nuclear *HMGA2* is regulated directly by miRNAs in HCT116 cells (Fig. 5). Recognition begins when *HMGA2* mRNA is associated with chromatin (Fig. 3C), but this binding does not affect transcription or splicing (Figs 6 and 7).

While beyond the scope of this report, we are investigating the molecular mechanism of miRNA-mediated regulation of *HMGA2* expression in cell nuclei. It is possible that the mechanism shares similarities with the known cytoplasmic mechanism miRNA-mediated translational repression. Previous work from our laboratory and others have demonstrated the association of members of the CCR4–NOT complex with AGO2 and TNRC6A in the nucleus by mass spectrometry and subsequently confirmed these associations by reciprocal immunoprecipitations of individual proteins [16, 57]. The similarity of protein complexes supports the hypothesis that the mechanisms of regulation will also be similar. Both mechanisms, cytoplasmic and nuclear, may contribute to the efficiency of miRNA-mediated gene silencing.

### Chimeric eCLIP: strengths, limitations, and innovation

Strengths: Chimeric eCLIP is a valuable approach for rapidly and unambiguously identifying miRNA:RNA target interactions that may be biologically relevant. Chimeric eCLIP was reproducible between replicate chimeric experiments done at different times. The complementary chimeric and nonchimeric data affords high confidence that the data reflects cellular interactions that may be biologically relevant.

The ability of chimeric eCLIP to provide data necessary for decisive candidate identification makes it a powerful approach to finding regulatory miRNA/target pairs in different cell types or tissues. Better prioritization of candidates is important because experimental validation is time-consuming. Ambiguous sequence results can lead to wasted effort or, even worse, misleading conclusions.

Limitations: Like all methods, chimeric eCLIP has limitations. One limitation more reflects the complexity of miRNA regulation than a shortcoming of the technology. Strong chimeric eCLIP data does not prove the existence of miRNA-mediated regulation. For example, we observe compelling evidence of AGO2 and *let-7* binding at the *MYC* gene but obtained no experimental evidence supporting miRNA-mediated gene regulation (Supplementary Fig. S10). The associations revealed by chimeric eCLIP data may reflect real interactions between miRNAs and RNA targets, but some interactions may not be efficient enough to produce significant repression of the target genes. That hypothesis would explain why we observe strong AGO2:*let7* association within the *MYC* 3′-UTR but were unable to obtain experimental evidence for on-target regulation of *MYC* expression by *let-7*.

Another limitation reflects the boundaries of the current technology. Well-expressed targets will be detected better than targets that are less well expressed. Transcripts that are not well expressed, are unstable, or are not accessible may not be detected with confidence or may not be detected at all. The power of sequencing will be directed absorbed by the highest expressed, most accessible transcripts.

Compounding this shortcoming, the need for a ligation step makes detection of chimeric reads relatively inefficient, further emphasizing recognition of well-expressed transcripts. Rather than detecting millions of reads like nonchimeric anti-AGO2 eCLIP, our chimeric eCLIP detected only tens of thousands of chimeric reads and may miss biologically important interactions. Those reads were adequate to detect the recognition of HMGA2 and a relatively small number of other genes, but we may be missing interactions with less expressed RNAs. For example, we are interested in the potential for binding to intronic RNA but may be missing less abundant target sequences within intronic RNA that affect splicing. We may also be missing targets within promoter RNAs that affect transcription.

Need for innovation: Technical innovation will be necessary to improve chimeric eCLIP and overcome these limitations. Methods are being developed that focus the power of chimeric eCLIP on specific genes, allowing much deeper insights at loci when miRNA-mediated control is suspected [48]. These methods may be especially important for investigating relatively lowly abundant sequences associated with chromatin, especially intronic sequences.

Specifically, we have a longstanding interest in probing the potential for endogenous miRNAs to regulate gene transcription and splicing. Our chimeric eCLIP reads did not reveal association of miRNAs within intronic or other noncoding RNAs, and our analysis at the *HMGA2* locus did not implicate regulation of transcription or splicing. One explanation is that these noncoding targets may be expressed at low levels too low for identification by chimeric eCLIP. Improved methods for targeted chimeric eCLIP [48] may alleviate this problem. Our nonchimeric data, however, revealed potential sites for interactions between AGO2 and introns and we are investigating these as candidate sites that may contribute to the regulation of splicing.

Alternatively, as we and others have shown, miRNAs and associated protein factors can shift within the cell depending on environmental conditions [22, 27, 50, 89–91]. miRNA-mediated regulation may become both observable and biologically important under a special set of cell growth of physiological conditions. Methods like chimeric eCLIP may be more productive when to be applied to those conditions where miRNA-mediated regulation is strongest.

## Conclusions

We observe that AGO2:miRNA complexes co-purify with chromatin in mammalian cells. miRNA-mediated gene silencing can occur in cell nuclei. While our data focusing on miRNA-mediated regulation of *HMGA2* expression offer a compelling model, the biological relevance of the binding of miRNAs to chromatin-associated RNAs and the molecular mechanism of gene repression will require additional research.

The most obvious associations between miRNAs and their targets in the chromatin fraction or whole nucleus occur within the 3′-UTR. That recognition within the 3′-UTR by *let-7* is responsible for remarkably efficient regulation of HMGA2 protein expression (Fig. 4B and D). While previous studies have shown that nuclear RNAs can regulate splicing or transcription and we observe recognition of chromatin-associated RNA, we do not observe miRNA-mediated control of either process at the *HMGA2* loci. More powerful, improved protocols for chimeric eCLIP or similarly innovative methods will be needed to better test the hypothesis that biologically active miRNAs can function by targeting intronic, promoter, or other less expressed RNAs.

Chimeric eCLIP is a powerful method for prioritizing gene candidates for miRNA-mediated regulation that merits wide use in studies that aim to identify miRNA:RNA target pairings. The data was robust and internally consistent, providing confident identification of major sites for binding by AGO2:miRNA complexes that justifies the time-consuming experimental validation necessary to link miRNA recognition to on-target regulation of a specific gene. While not all targets will be identified, the methodology optimizes the likelihood that at least some outstanding candidates will be defined. The identification of nuclear and cytoplasmic RNA targets for miRNA and how recognition in both compartments cooperate to shape gene regulation may impact the understanding of human cell biology and drug discovery.

## Supplementary Material

gkaf800_Supplemental_File

## Data Availability

All data described are contained within the article, the Supplementary Data, or have been deposited in public databases under the accession number GSE297116.
