# Supplementary material for: Nuclear Argonaute:miRNA complexes recognize target sequences within chromatin-associated RNA and silence gene expression
Source: Nucleic Acids Res. 2025 Aug 27;53(16):gkaf800. doi: 10.1093/nar/gkaf800 (PMC12390760; doi:10.1093/nar/gkaf800)
Supplement: gkaf800_Supplemental_File [file gkaf800_supplemental_file.pdf]

SUPPLEMENTARY INFORMATION

Gene	Feature targeted	Forward primer (5' to 3')	Reverse primer (5' to 3')
HMGA2	mature mRNA	GTTGGTCGCAGCTACATAAGA	ACTCAAAGGAACAGGGAGAAAG
HMGA2	pre-mRNA	AGCAGCCCACACAGTATAAC	GACTCTTGTGAGGATGTCTCTTC
MYC	mature mRNA	CTGAGGAGGAACAAGAAGATGAG	TGTGAGGAGGTTTGCTGTG
MYC	pre-mRNA	GTCCAAAGCCTCATTAAGTCTTAGGTA	CAACTTCCCAGGATAGGACATTG
ZFP36	mature mRNA	CATGGATCTGACTGCCATCTAC	ACTCAGTCCCTCCATGGTC
ZFP36L1	mature mRNA	GAACGCACAGGATGACCA	CACTGGGAGCACTATAGTTGAG
HMGA2	Promoter (ChIP)	ACTTGGCAGAAAGAGAGTTCTCAGGC	TTCTCCAGGAAAGACTAGAGGCAACC
GAPDH	Promoter (ChIP)	TACTAGCGGTTTTACGGGCG	TCGAACAGGAGCAGAGAGCGA
HMGA2	Exon 2 inclusion	AGCAGCAAGAACCAACCG	CTTTTGAGCTGCTTTAGAGGGAC
HMGA2	Exon 2 skipping	AGCCGTCCACTTCAGCCC	CTTCTGCTTTTTGCTGCTGCTT
HMGA2	Exon 3 inclusion	CAGCTCAAAAGAAAGCAGAAGC	CCATTTCCTAGGTCTGCCTC
HMGA2	Exon 3 skipping	GAACCAACCGGTGAGCCC	CTTGTTGTGGCTTTTGAGCTG
HMGA2	Exon 4 inclusion	TAGGAAATGGCCACAACAAG	CTGAGCAGGCTTCTTCTGA
HMGA2	Exon 4 skipping	AAAGCAGAAGCCACTGGAG	TTCAGTTTCCTCCCATTTCCTAG
HMGA2	Exon 4-Exon-5, full mRNA	CCTGCTCAGGAGGAAACTG	CTAGTCCTCTTCGGCAGAC
TBP	Exon 4 inclusion	CGCAGCTGCAAAATATTGTATC	CTTGGGATTATATTCGGCGTTTC
TBP	Exon 4 skipping	CACTGTATCCCTCCCCCATG	CAGCAAACCGTGCAGCTG
TBP	Exon 7-Exon 8, full mRNA	TTGTATTAACAGGTGCTAAAGTCA	TTACGTCGTCTTCCTGAATCC

Supplementary Table 1. Primers for qPCR used in this study

ID	Sequence	Note
NC	QIAGEN, 339136 Y100199006-DDA	Negative control, LNA
Let-7 in	QIAGEN, 339160 YFI0450006	Let-7 family, LNA inhibitor
miR29 in	QIAGEN, 339160 YFI0450039	miR29 LNA inhibitor
Let-7a	5’-UGAGGUAGUAGGUUGUAUAGUU (5p)<br>5’-CUAUACAAUCUACUGUCUUUC (3p)	hsa-let-7a
Let-7mm	5’-UGCAAUAAUAGGUUGUAUAGUU (5p)<br>5’-CUAUACAAUCUACUGUCUUUC (3p)	hsa-let-7a seed mismatch control
miR27	5’-AGGGCUUAGCUGCUUGUGAGCA (5p)<br>5’-UUCACAGUGGCUAAGUUCCGC (3p)	hsa-miR27
CM	5’-GCUAUACCAGCGUCGUCAUdTdT<br>5’-AUGACGACGCUGGUAUAGCdTdT	Non-complimentary control

Supplementary Table 2. miRNA inhibitors and mimics used in this study

Sample Name	Total reads	Final non-chimeric reads	Ago2 peaks	Reproducible Ago2 peaks	Final chimeric reads	miRNA:mRNA chimeric reads	miRNA:mRNA reads in miR-first orientation	% of miRNA:mRNA reads in miR-first orientation	Chimeric peaks	Reproducible chimeric peaks
WT Whole Cell 1	60,325,177	12,948,646	2,219	1,567	97,076	69,768	66,585	95%	764	359
WT Whole Cell 2	58,114,574	15,513,844	3,540		107,386	71,962	70,470	98%	585	
NLS-Ago2 Whole Cell 1	62,413,068	15,624,859	2,611	1,338	81,000	52,150	51,169	98%	401	211
NLS-Ago2 Whole Cell 2	59,605,781	16,100,029	2,269		73,545	47,672	46,320	97%	361	
WT Cytoplasm 1	60,283,679	9,534,156	1,236	525	175,318	128,553	126,552	98%	2,111	730
WT Cytoplasm 2	61,415,583	11,695,461	1,194		164,493	102,072	89,708	88%	1,066	
WT Chromatin 1	56,461,192	16,592,727	1,911	1,173	13,805	4,447	3,938	89%	15	11
WT Chromatin 2	58,883,592	14,913,388	2,466		15,048	5,086	4,219	83%	49	

Supplementary Table 3. Quality control metrics for all chimeric eCLIP samples

A

Cytoplasm					
**Gene**	**Strand**	**Gene expression FC (Drosha-/- vs. WT)**	**Ago2 chimeric peaks with miRNA seed match in 3' UTR**	**Number of unique miRNAs**	**All miRNAs**
HMGA2	+	5.0	26	15	miR-20a-5p, miR-93-5p, miR-106b-5p, miR-29a-3p, let-7a-5p, let-7d-5p, let-7f-5p, miR-17-5p, let-7b-5p, let-7g-5p, miR-16-5p, miR-33b-5p, miR-15a-5p, miR-15b-5p
MYC	+	-8.8	7	5	let-7a-5p, let-7b-5p, let-7d-5p, let-7e-5p, miR-320a-3p
TNFRSF12A	+	1.4	2	2	miR-19b-3p, miR-19a-3p
ZFP36	+	-2.3	6	6	miR-222-3p, miR-31-5p, miR-27a-3p, miR-200c-3p, miR-29b-3p, miR-29a-3p
ZFP36L1	-	3.4	8	7	miR-222-3p, let-7a-5p, miR-23a-3p, miR-31-5p, miR-18a-5p, miR-29b-3p, miR-29a-3p

B

Nucleus					
**Gene**	**Strand**	**Gene expression FC (Drosha-/- vs. WT)**	**Ago2 chimeric peaks with miRNA seed match in 3' UTR**	**Number of unique miRNAs**	**All miRNAs**
HMGA2	+	5.0	8	8	let-7a-5p, let-7d-5p, miR-106b-5p, miR-20a-5p, miR-17-5p, let-7b-5p, let-7f-5p, miR-16-5p
MYC	+	-8.8	2	2	let-7b-5p, let-7d-5p
TNFRSF12A	+	1.4	2	2	miR-19b-3p, miR-19a-3p
ZFP36	+	-2.3	2	2	miR-31-5p, miR-27a-3p
ZFP36L1	-	3.4	1	1	miR-29b-3p

C

Chromatin					
**Gene**	**Strand**	**Gene expression FC (Drosha-/- vs. WT)**	**Ago2 chimeric peaks with miRNA seed match in 3' UTR**	**Number of unique miRNAs**	**All miRNAs**
HMGA2	+	5.0	8	5	miR-16-5p, let-7d-5p, let-7a-5p, let-7f-5p, let-7b-5p
MYC	+	-8.8	1	1	let-7b-5p
TNFRSF12A	+	1.4	2	2	miR-19b-3p, miR-19a-3p

Supplementary Table 4. Argonaute 2 chimeric binding sites in gene 3'UTR identified in the (A) cytoplasm, (B) nucleus and (C) chromatin. Genes were identified based on number of chimeric binding peaks per gene and conservation of binding peaks across subcellular fractions. Peaks were defined to have a p-value<0.001 and log2FC>3 (IP vs. input).

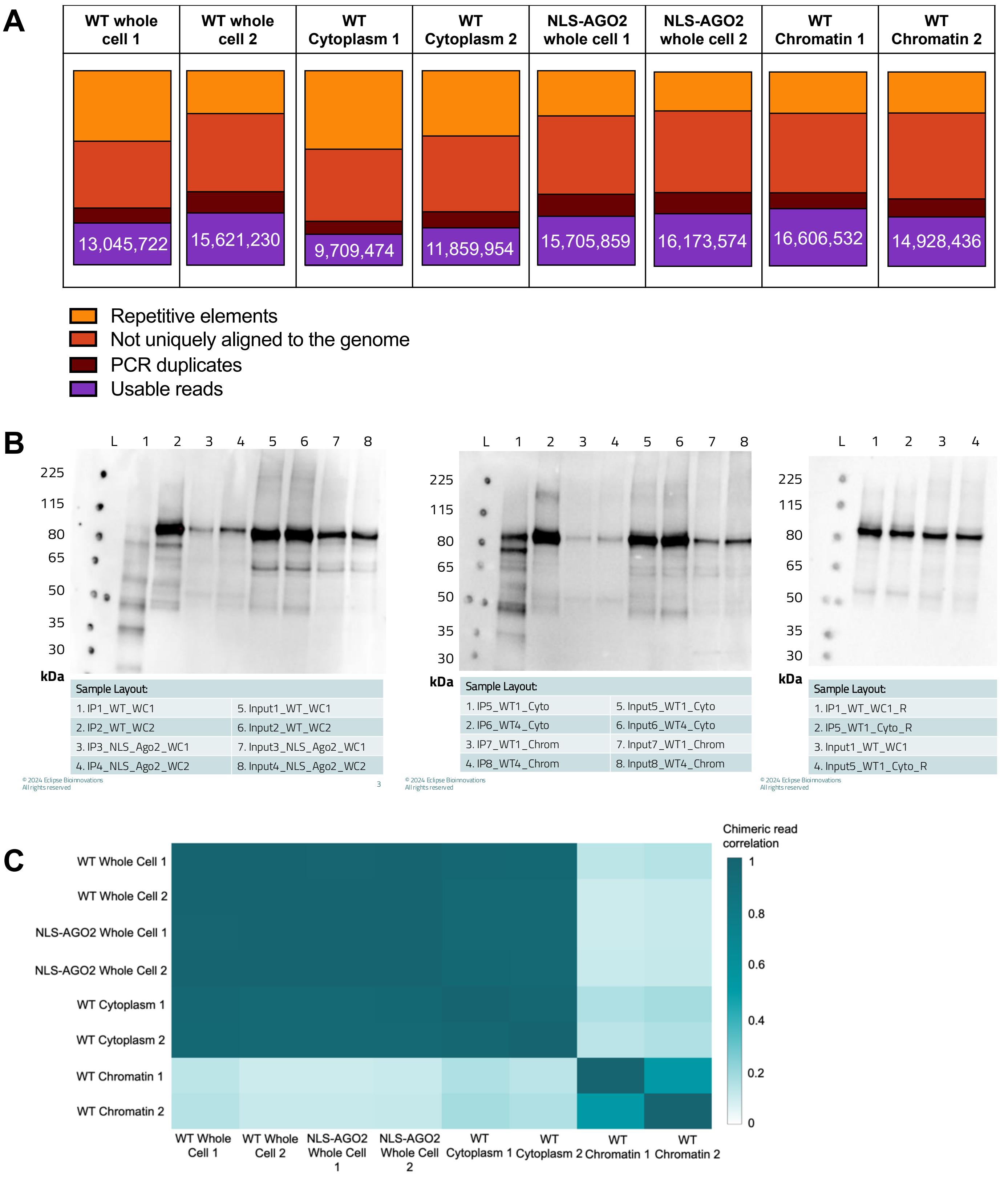

Supplementary figure 1. AGO2 chimeric eCLIP quality control. **(A)** Total reads from each replicate of chimeric eCLIP aligning as repetitive elements, not uniquely aligning to the genome, PCR duplicates, and usable reads. **(B)** Western blot probing for AGO2, validating input and IP samples used in chimeric eCLIP. **(C)** Correlation plot of all chimeric eCLIP samples

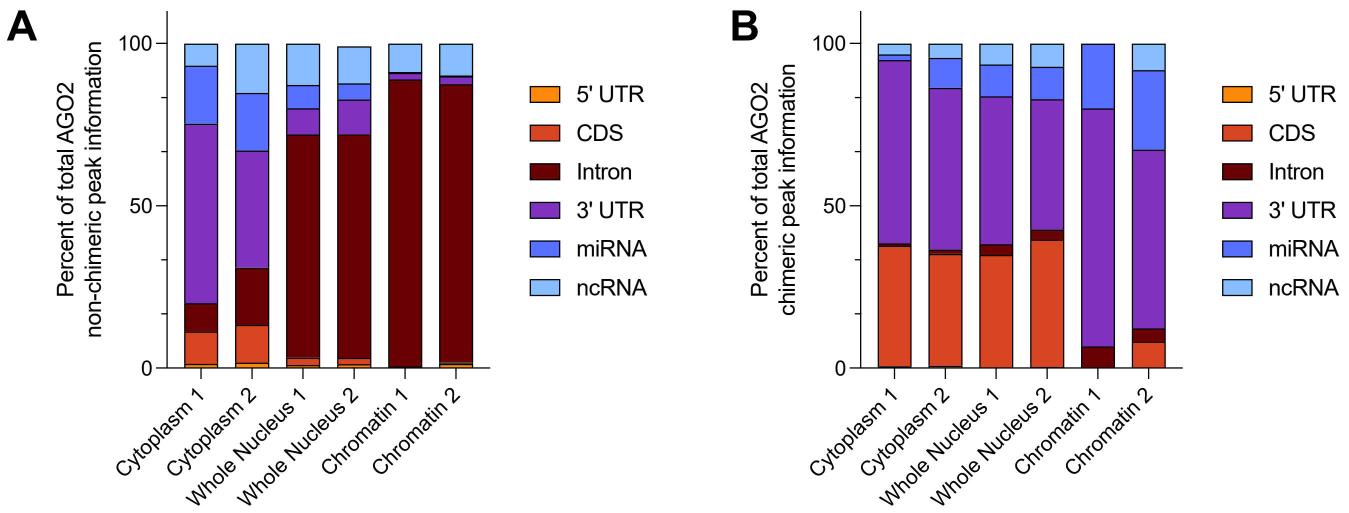

Supplementary figure 2. Distribution of AGO2 non-chimeric and chimeric peak binding. **(A)** Individual replicate data from figure 2B, showing percentage of non-chimeric AGO2 binding across genomic features. **(B)** Individual replicate data from figure 2C, showing percentage of chimeric AGO2 binding across genomic features. Whole nucleus refers to NLS-AGO2 whole cell samples.

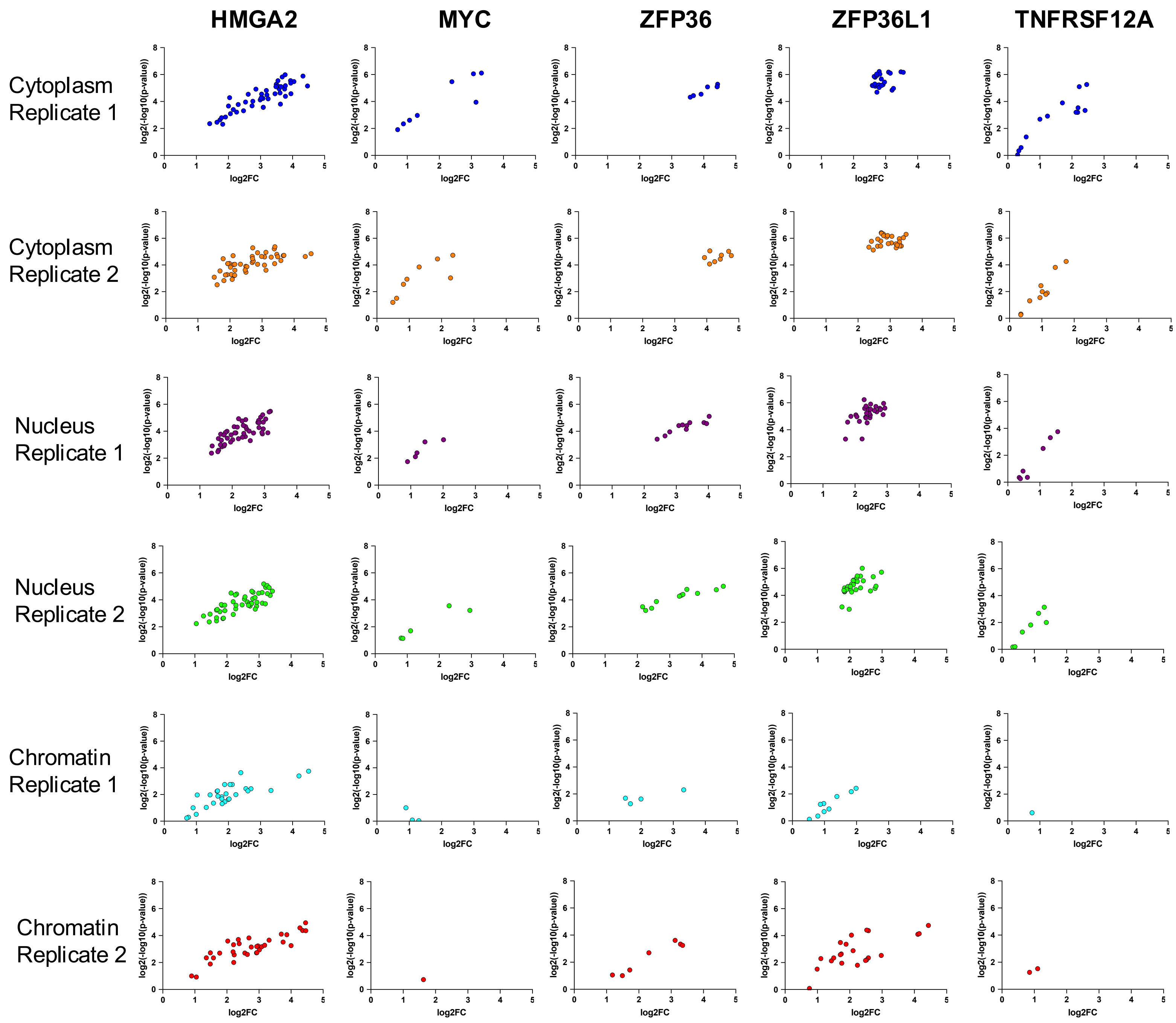

Supplementary figure 3. Strength of individual non-chimeric AGO2 peaks in the 3'UTR of genes. All non-chimeric AGO2 peaks in the 3'UTR of the five genes (from Supplementary Table 3) are plotted as log2FC (IP vs. input) vs. peak significance (log2(-log10(p-value)), with each dot representing a single AGO2 peak. Following IP and sequencing, peaks were identified following read alignment to the genome and identification using CLIPper. Whole nucleus refers to NLS-AGO2 whole cell samples.

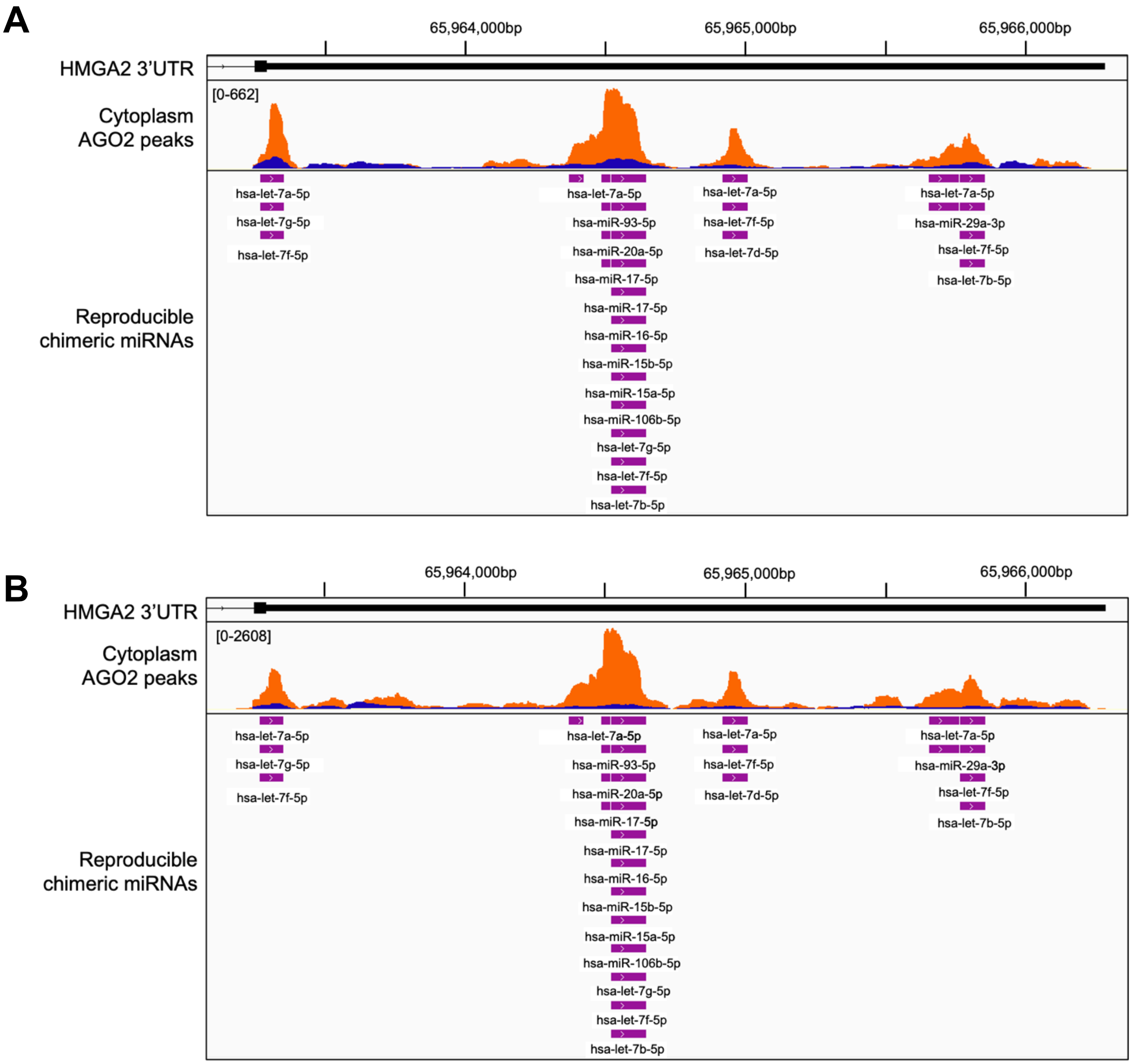

Supplementary figure 4. HMGA2 is bound by AGO2 in all compartments of the cell. IGV browser images of chimeric eCLIP reads from **(A)** replicate 1 and **(B)** replicate 2 within the 3'UTR of HMGA2 in the cytoplasm. Chimeric AGO2 peaks are in orange and non-chimeric AGO2 peaks are overlayed in navy blue. HMGA2 is located on chromosome 12 (chr12). Peak height is defined as read density in reads per million (RPM). All AGO2 peaks are reproducible and meet the following criteria: log2FC>3 (IP vs. input) at least 3 reads per peak.

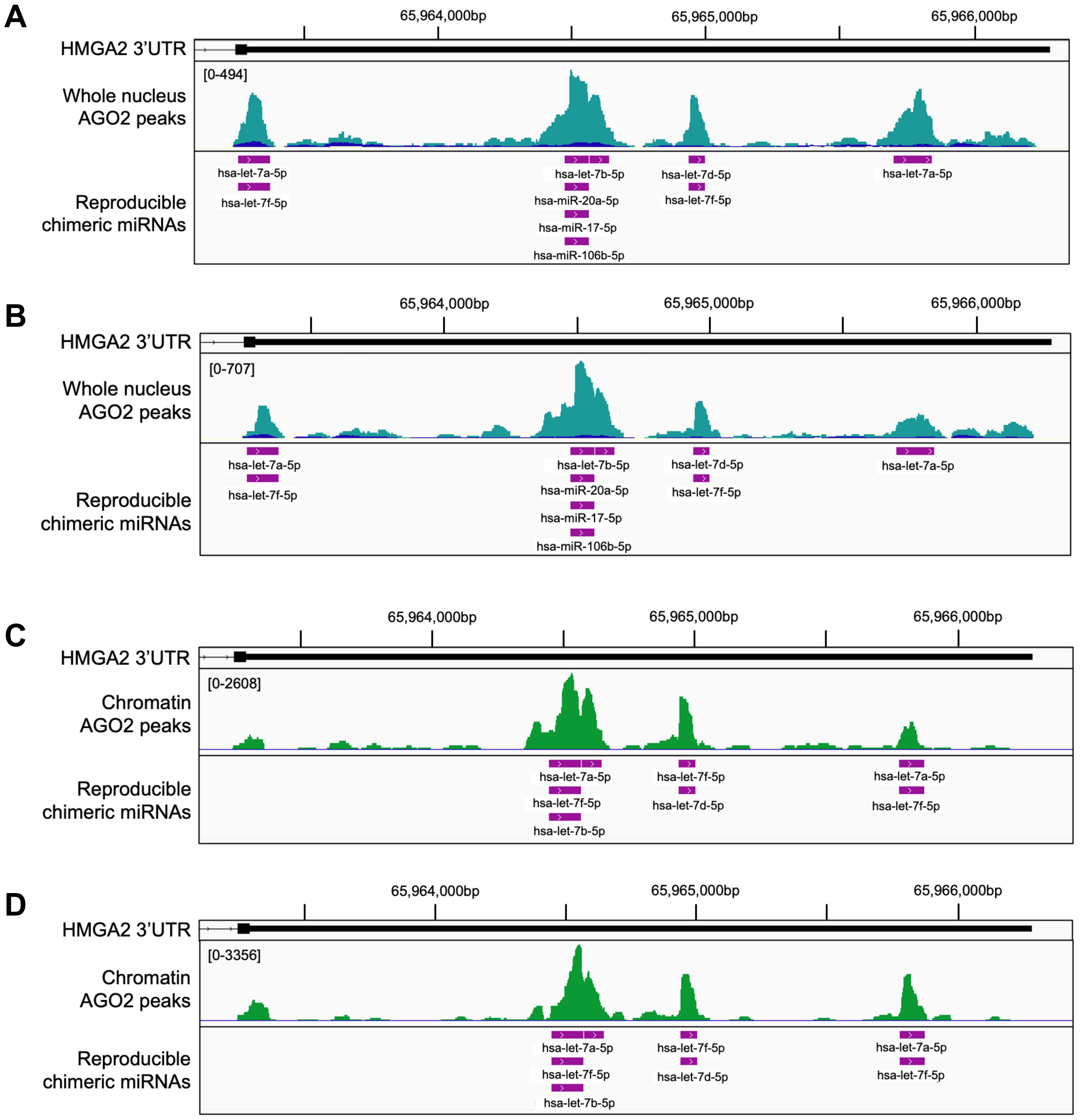

Supplementary figure 5. HMGA2 is bound by AGO2 in all compartments of the cell. IGV browser images of chimeric eCLIP reads within the 3'UTR of HMGA2 in the **(AB)** nucleus (NLS-AGO2 whole cell) and **(CD)** chromatin. Chimeric AGO2 peaks are in teal (nucleus) and green (chromatin) and non-chimeric AGO2 peaks are overlayed in navy blue. HMGA2 is located on chromosome 12 (chr12). Peak height is defined as read density in reads per million (RPM). All AGO2 peaks are reproducible and meet the following criteria: log2FC>3 (IP vs. input) at least 3 reads per peak.

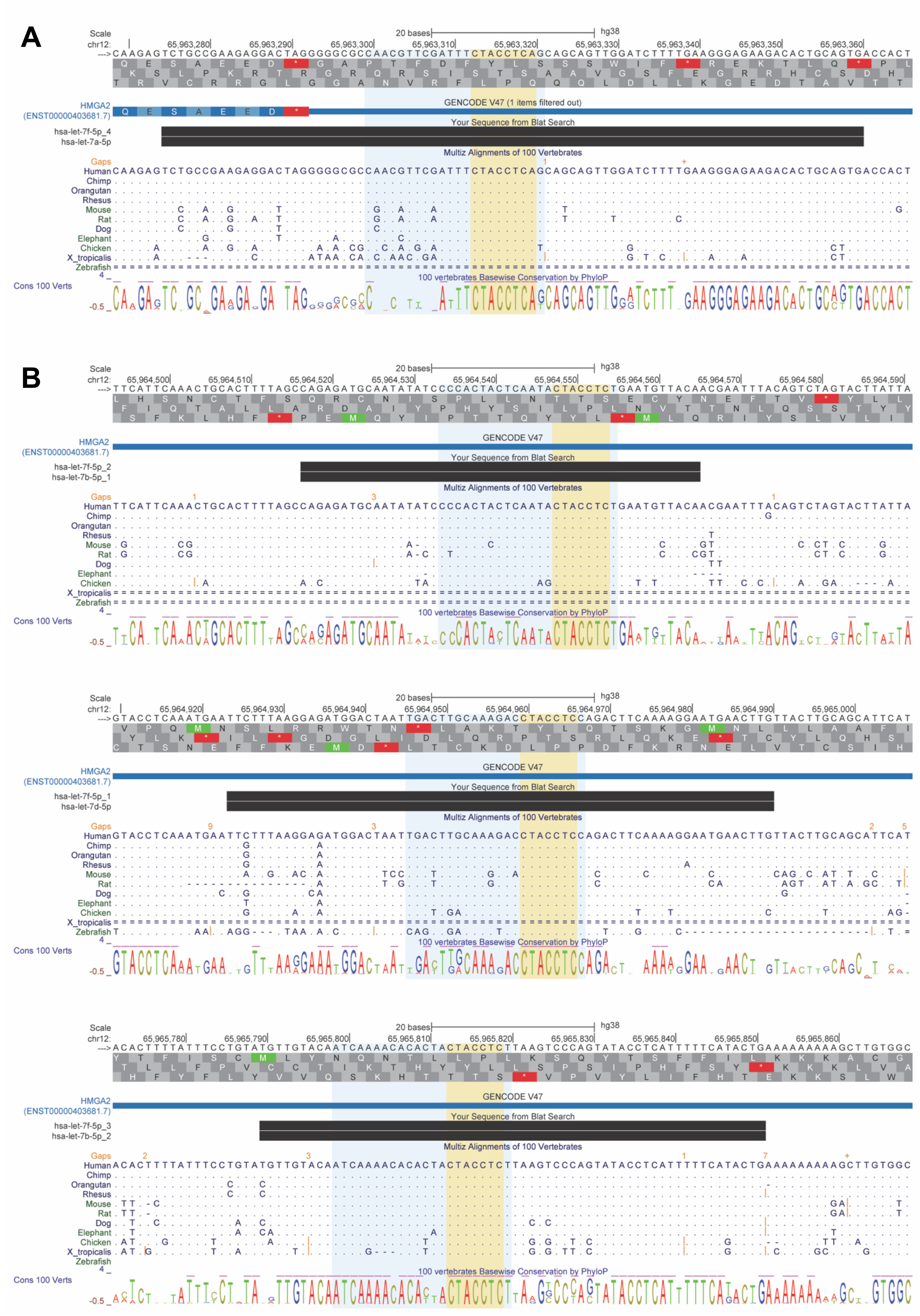
A
B
Scale
20 bases
hg38
chr12:
HMGA2
(ENST00000403681.7)
GENCODE V47 (1 items filtered out)
GENCODE V47
Your Sequence from Blat Search
hsa-let-7f-5p_4
hsa-let-7a-5p
hsa-let-7f-5p_2
hsa-let-7b-5p_1
hsa-let-7f-5p_1
hsa-let-7d-5p
hsa-let-7f-5p_3
hsa-let-7b-5p_2
Multiz Alignments of 100 Vertebrates
Gaps
Human
Chimp
Orangutan
Rhesus
Mouse
Rat
Dog
Elephant
Chicken
X_tropicalis
Zebrafish
Cons 100 Verts
100 vertebrates Basewise Conservation by PhyloP

Supplementary Figure 6. The seed sequence of Let-7 miRNAs from this study are strongly conserved across species within the 3′ UTR of *HMGA2*. (A) Alignment and comparison of let-7 miRNAs with perfect matches to seed sequences. **(B)** Alignment and comparison of let-7 miRNAs with imperfect matches to seed sequences. The genomic coordinates, followed by the reading frames for canonical and alternative amino acid sequences (top and bottom, respectively), including all nucleotides shown in (A) and (B) for *HMGA2*, NM_003483.6 (ENSG00000149948.14), corresponds to the sense strand of the gene. The span of the entire miRNA sequence is indicated in light blue whereas the seed sequence is indicated in yellow. The canonical full-length transcript for *HMGA2* of the human genome is indicated in blue under the GENCODE v47 track. The 3′ UTR is indicated by a thin box whereas a thicker box with amino acid symbols corresponds to the coding sequence. Chimeric eCLIP sequence contexts aligned to the human genome is indicated in black boxes under the BLAT Search track. Multiple alignment and comparison of genomes are indicated by their species under the Multiz Alignments of 100 Vertebrates track, in addition to their basewise conservation across species under the 100 Vertebrates Basewise Conservation by PhlyoP track. Under the Multiz Alignment track, each dot for a given species at a given position represents an identical nucleotide match to the human reference genome. Each single or double line for a given species at a given position corresponds to no nucleotide(s) or unalignable nucleotides to the reference, respectively. Gold line and text for a given species corresponds to gaps in the region of “N” nucleotide length (a gap of “+” indicates a larger than mappable gap on the UCSC Genome Browser viewer). Vertical blue bars and green square brackets correspond to genomic breaks (e.g., such is the case for Zebrafish; the genomic break occurs at GRCh38/hg38 coordinates chr12:65,965,479-65,965,479, resulting in a proceeding empty row observed in the last snapshot of (B)).

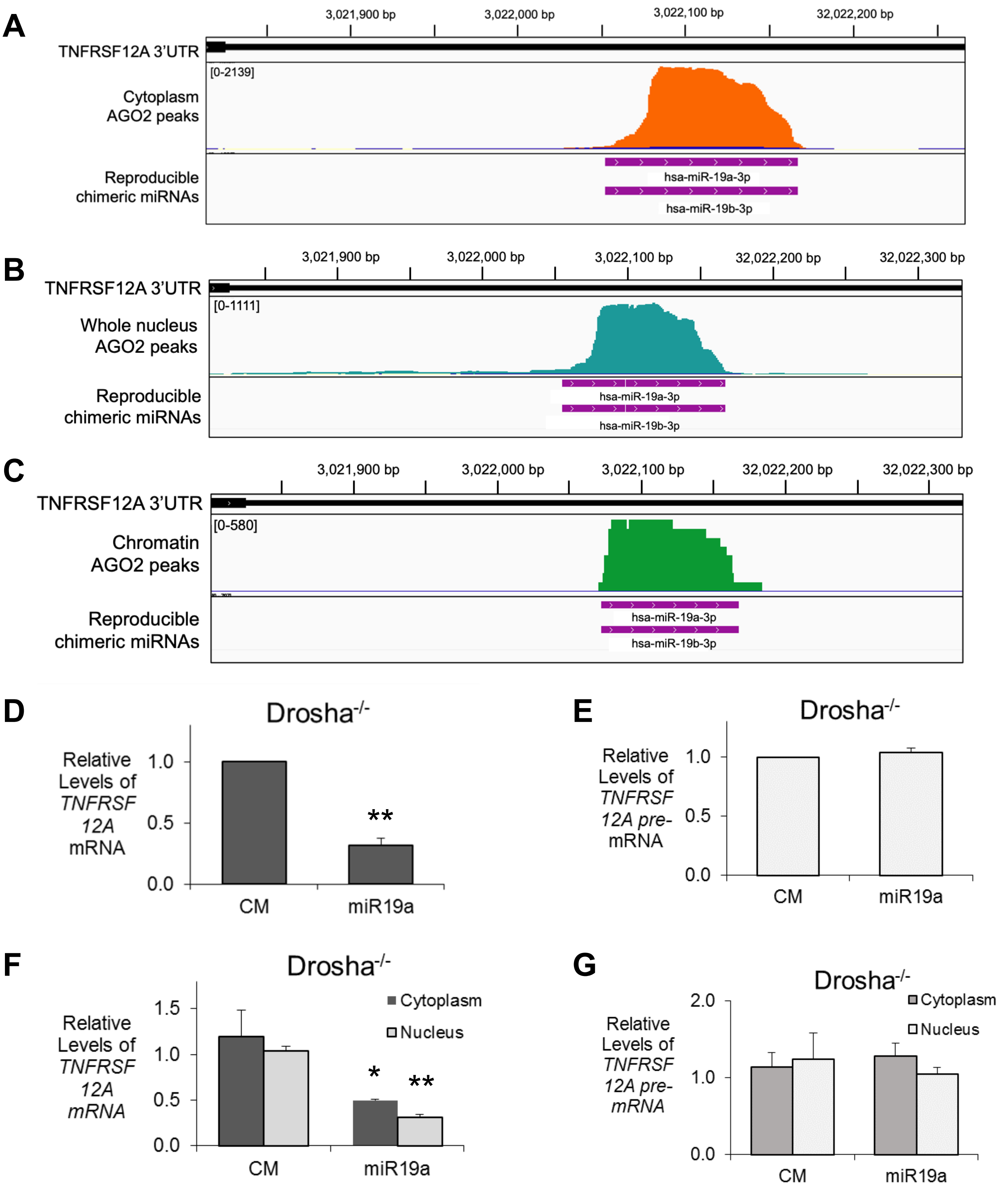

Supplementary Figure 7. Evidence that *TNFRSF12A* mature mRNA is a miRISC target that may be regulated by *miR-19a*. IGV browser views (replicate 1) of chimeric eCLIP reads within the *TNFRSF12A* 3'UTR in the **(A)** cytoplasm, **(B)** nucleus (NLS-AGO2 whole cell), and **(C)** chromatin. Mature *TNFRSF12A* mRNA levels in (**D)** *DROSHA-/-* cells transfected with duplex RNA control (CM) or *miR-19a* miRNA mimic, whole cell RNA levels. **(E)** *TNFRSF12A* pre-mRNA levels in *DROSHA*-/- cells transfected with duplex RNA CM control or *miR-19a* miRNA mimic are unchanged. **(F)** Mature mRNA and **(G)** pre-mRNA levels of *TNFRSF12A* in the cytoplasm and nucleus of *Drosha-/-* cells transfected with control duplex RNA CM or miR-19a miRNA mimic. Significance denoted as *p<0.05; **p<0.01; ***p<0.001.

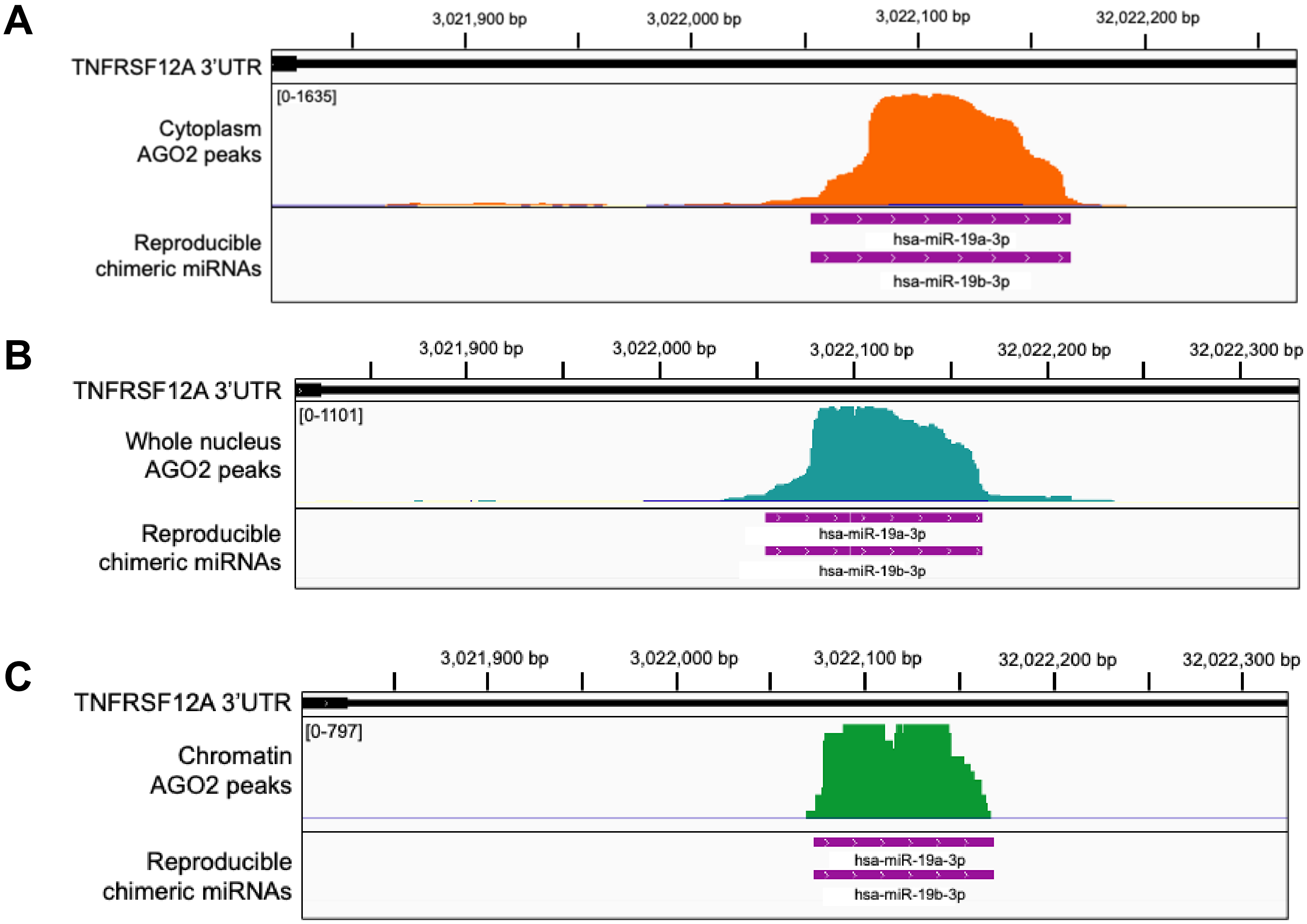

Supplementary Figure 8. Binding of AGO2 and miRNAs to *TNFRSF12A* RNA in HCT116 cells is reproducible. In contrast to **Figure 3**, chimeric and nonchimeric data is set to the same Y-axis scale. Replicate IGV browser images of chimeric eCLIP reads within the 3'UTR of TNFRSF12A in the **(A)** cytoplasm, **(B)** nucleus (NLS-AGO2 whole cell), and **(C)** chromatin. Chimeric AGO2 peaks are in orange (cytoplasm), teal (nucleus), and green (chromatin) and non-chimeric AGO2 peaks are overlayed in navy blue. Peak height is defined as read density in reads per million (RPM). All AGO2 peaks are reproducible and meet the following criteria: log2FC>3 (IP vs. input) at least 3 reads per peak.

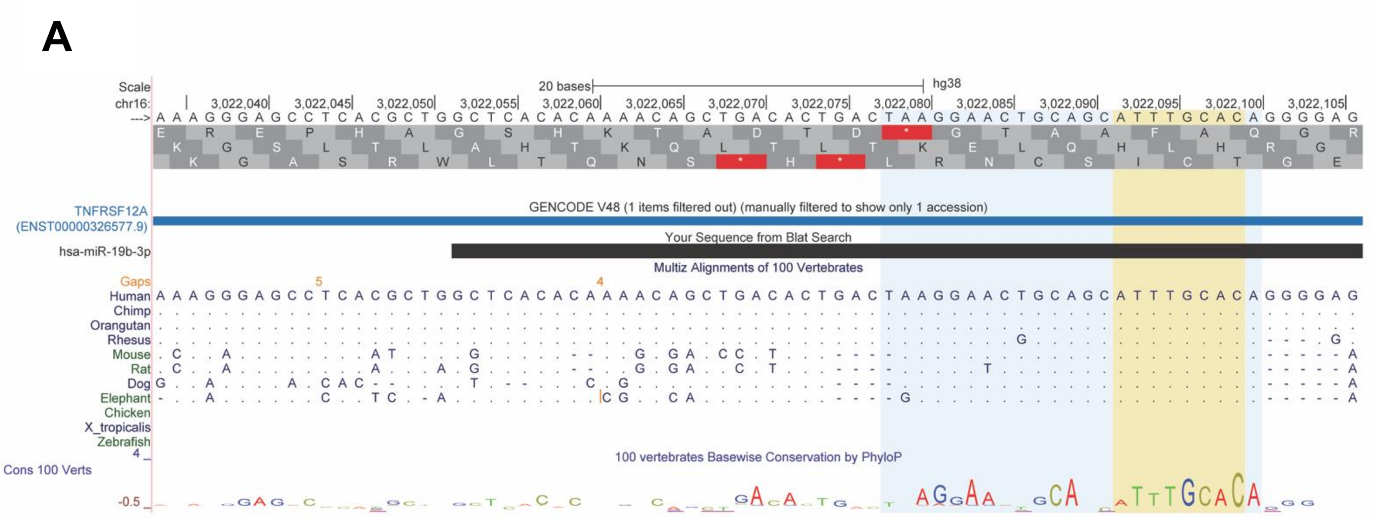

Supplementary figure 9. (A) Alignment and comparison of miR-19b with a perfect match to its seed sequence. The genomic coordinates, canonical (top) and alternative (bottom two) reading frames with amino acid symbols, and all nucleotides shown in (A) for *TNFRSF12A*, NM_016639.3 (ENSG00000006327.14), corresponds to the sense strand of the gene. The span of the entire miRNA sequence is indicated in light blue whereas the seed sequence is indicated in yellow. The canonical full-length transcript for *TNFRSF12A* of the human genome is indicated in blue under the GENCODE v48 track. The 3′ UTR is indicated by a thin box whereas a thicker box with amino acid symbols corresponds to the coding sequence. Chimeric eCLIP sequence contexts aligned to the human genome is indicated in black boxes under the BLAT Search track. Multiple alignment and comparison of genomes are indicated by their species under the Multiz Alignments of 100 Vertebrates track, in addition to their basewise conservation across species under the 100 Vertebrates Basewise Conservation by PhlyoP track. Under the Multiz Alignment track, each dot for a given species at a given position represents an identical nucleotide match to the human reference genome. Each single or double line for a given species at a given position corresponds to no nucleotide(s) or unalignable nucleotides to the reference, respectively. Gold line and text for a given species corresponds to gaps in the region of “N” nucleotide length (a gap of “+” indicates a larger than mappable gap on the UCSC Genome Browser viewer). Vertical blue bars and green square brackets correspond to genomic breaks.

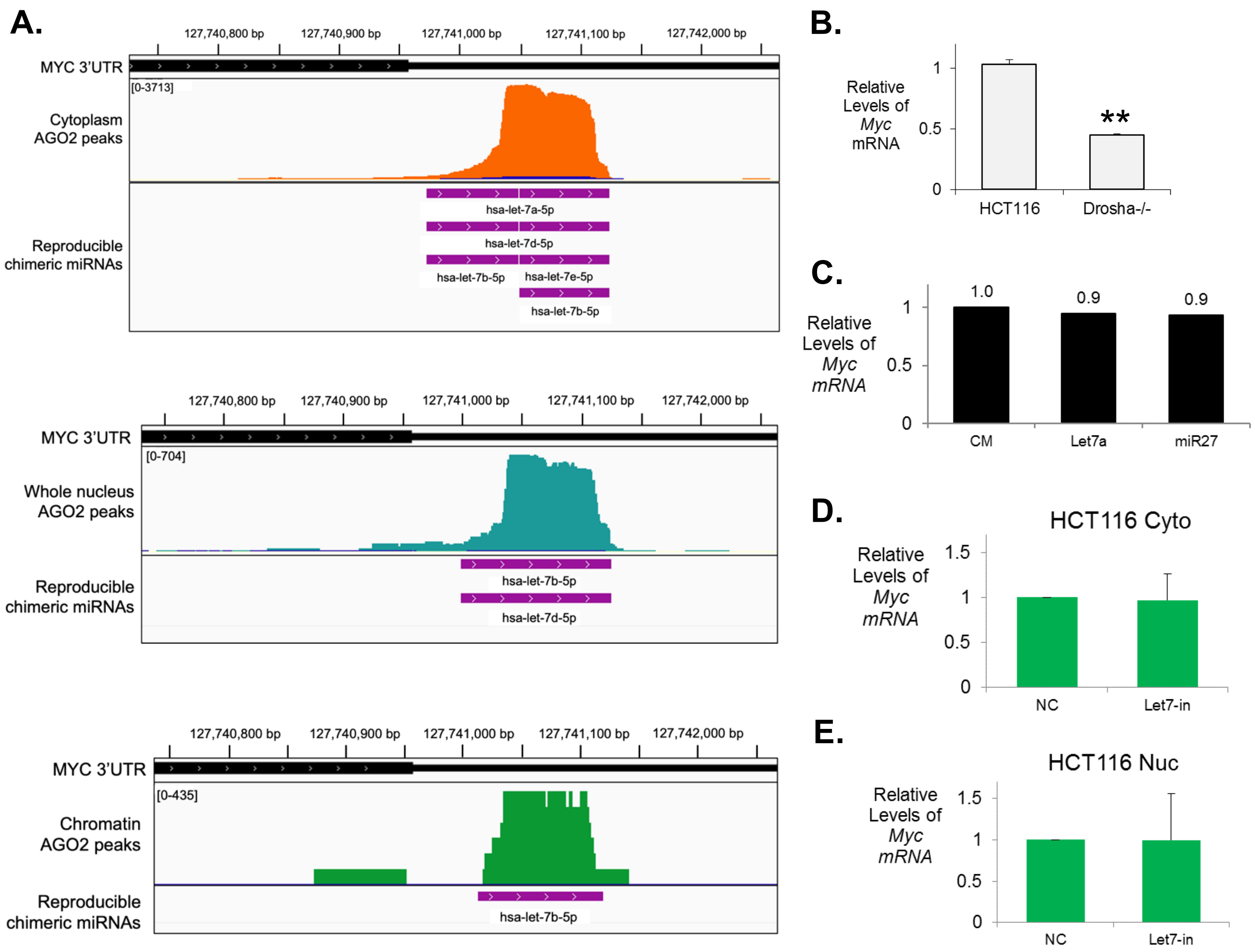

Supplementary figure 10. *MYC* is a miRISC target that does not appear to be regulated by the *let-7* family of miRNAs. (**A**) Representative IGV browser views of chimeric eCLIP reads within the *MYC* 3'UTR in the cytoplasm (top), nucleus (middle), and chromatin (bottom). Mature MYC mRNA levels in (**B**) wildtype and *DROSHA*-/- cells, (**C**) *DROSHA*-/- cells transfected *with let-7a* miRNA mimics, and WT (**D**) cytoplasm and (**E**) nucleus fractions following treatment with *let-7* family miRNA inhibitors. Values are plotted as the average +/- SD. Significance denoted as *p<0.05; **p<0.01; ***p<0.001. Whole nucleus refers to NLS-AGO2 whole cell samples.

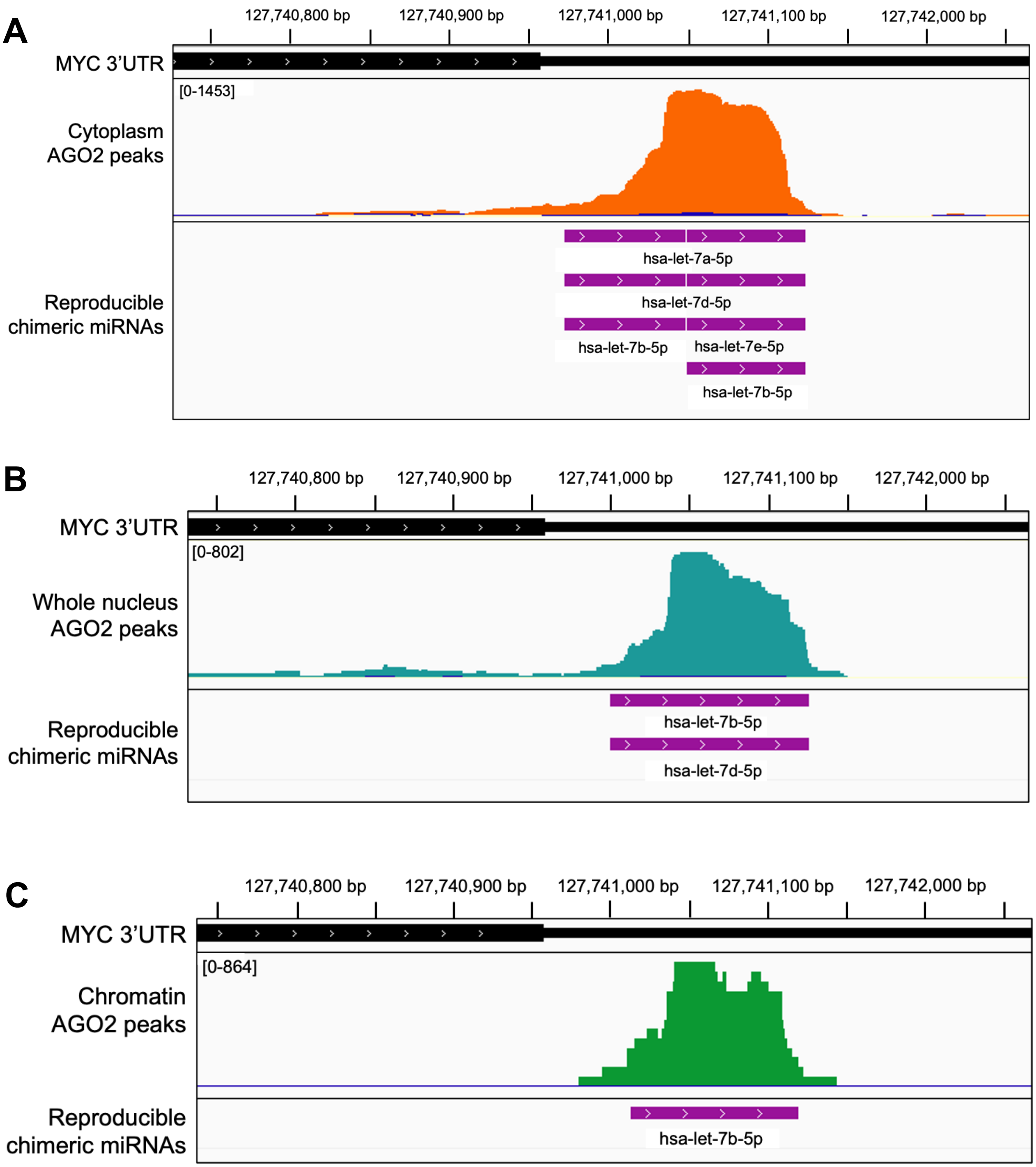

Supplementary figure 11. *MYC* is bound by AGO2 in all compartments of the cell. Replicate IGV browser images of chimeric eCLIP reads within the 3'UTR of *MYC* in the **(A)** cytoplasm, **(B)** nucleus (NLS-AGO2 whole cell), and **(C)** chromatin. Chimeric AGO2 peaks are in orange (cytoplasm), teal (nucleus), and green (chromatin) and non-chimeric AGO2 peaks are overlayed in navy blue. Peak height is defined as read density in reads per million (RPM). All AGO2 peaks are reproducible and meet the following criteria: log2FC>3 (IP vs. input) at least 3 reads per peak.

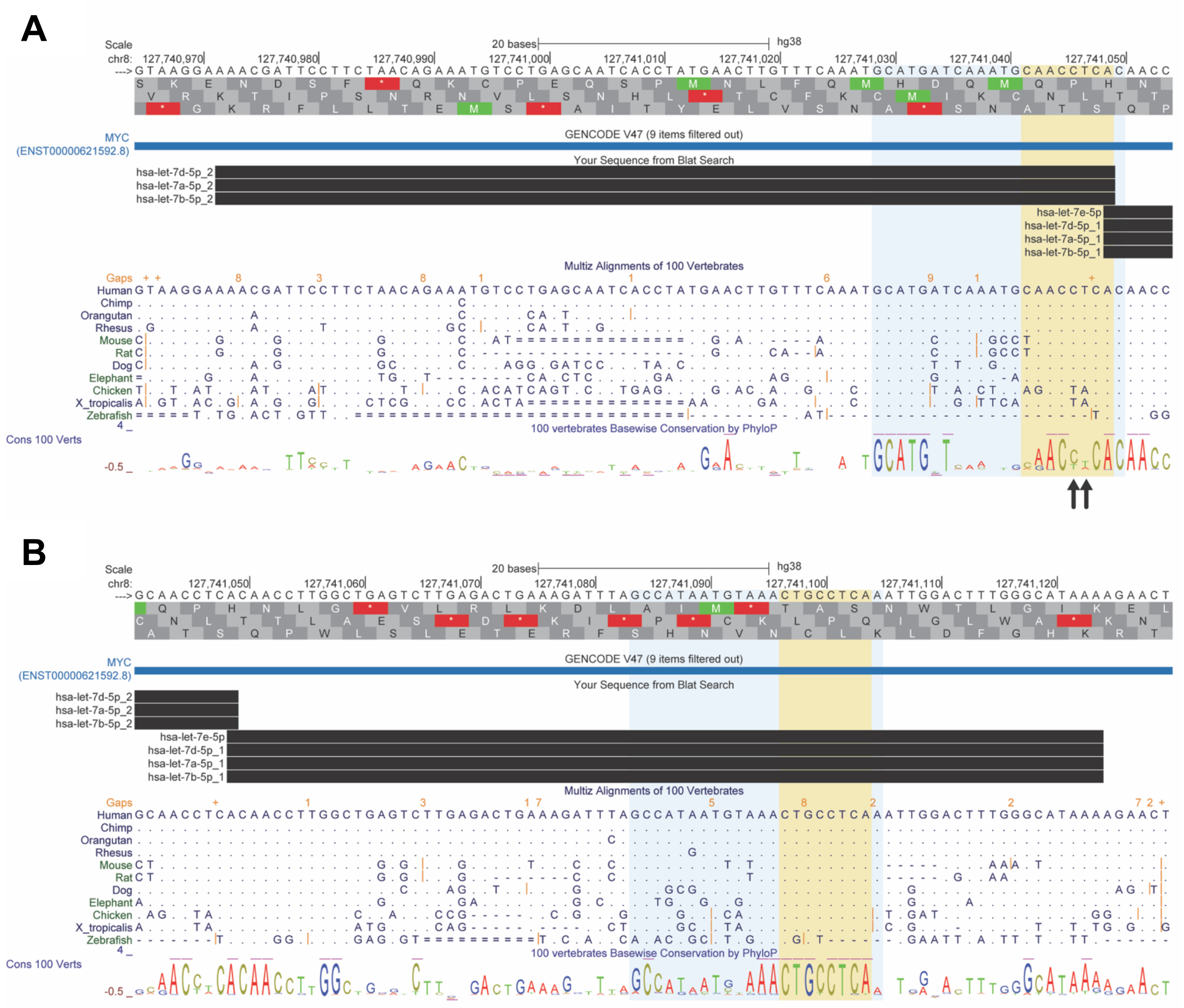

Supplementary Figure 12. The seed sequence of Let-7 miRNAs from this study are also strongly conserved across species within the 3′ UTR of *MYC*. (A) Alignment and comparison of *let-7* miRNAs with imperfect matches to seed sequences that exhibit relatively strong conservation. **(B)** Alignment and comparison of let-7 miRNAs with imperfect matches to seed sequences that exhibit strong conservation. The genomic coordinates, followed by the reading frames for canonical and alternative amino acid sequences (top and bottom, respectively), including all nucleotides shown in (A) and (B) for *MYC*, NM_002467.6 (ENSG00000136997.22), corresponds to the sense strand of the gene. The span of the entire miRNA sequence is indicated in light blue whereas the seed sequence is indicated in yellow. The canonical full-length transcript for *MYC* of the human genome is indicated in blue under the GENCODE v47 track. The 3′ UTR is indicated by a thin box whereas a thicker box with amino acid symbols corresponds to the coding sequence. Chimeric eCLIP sequence contexts aligned to the human genome is indicated in black boxes under the BLAT Search track. Multiple alignment and comparison of genomes are indicated by their species under the Multiz Alignments of 100 Vertebrates track, in addition to their basewise conservation across species under the 100 Vertebrates Basewise Conservation by PhlyoP track. Under the Multiz Alignment track, each dot for a given species at a given position represents an identical nucleotide match to the human reference genome. Each single or double line for a given species at a given position corresponds to no nucleotide(s) or unalignable nucleotides to the reference, respectively. Gold line and text for a given species corresponds to gaps in the region of “N” nucleotide length (a gap of “+” indicates a larger than mappable gap on the UCSC Genome Browser viewer). Vertical blue bars and green square brackets correspond to genomic breaks.

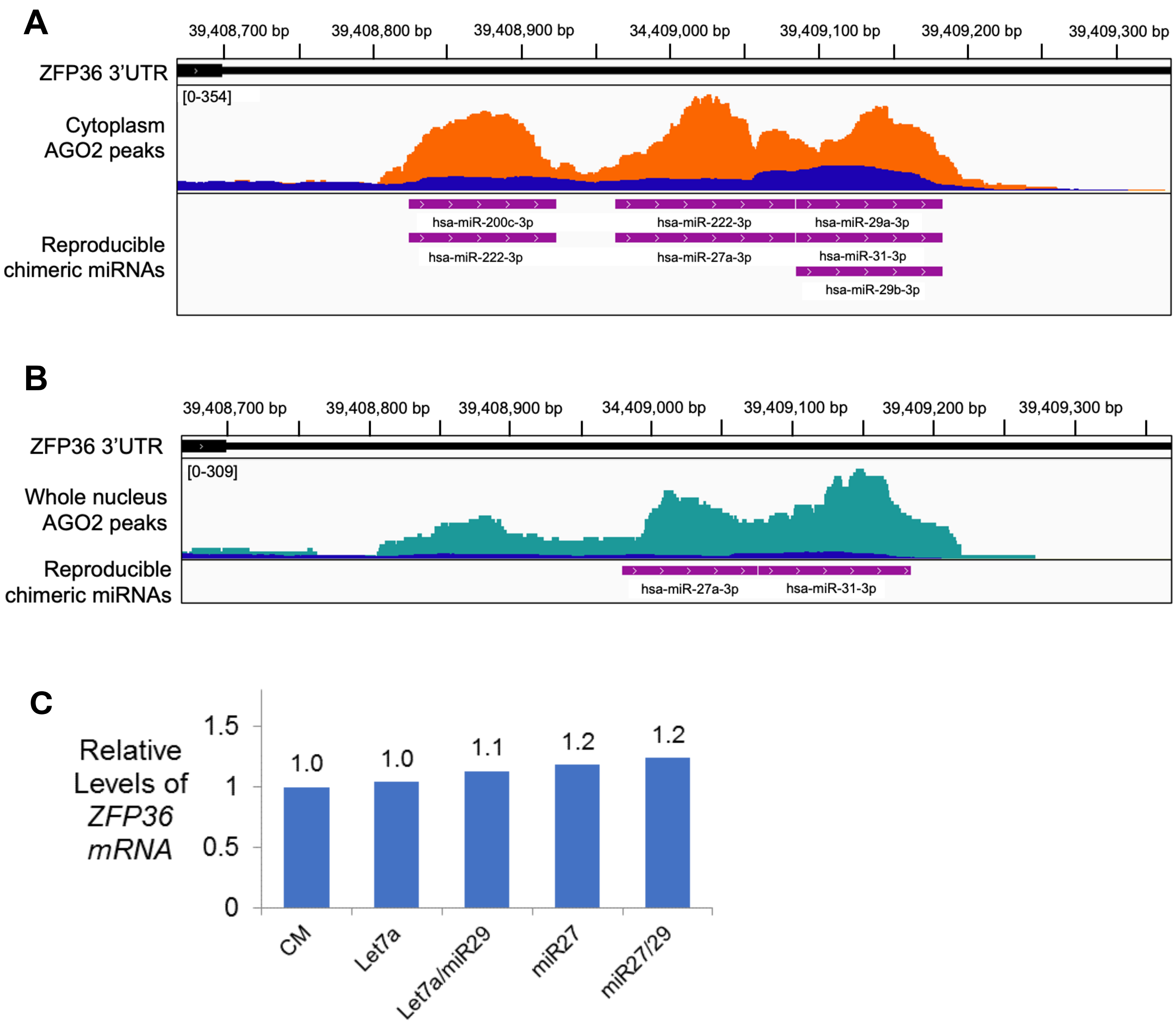

Supplementary figure 13. *ZFP36* does not seem to be simply regulated by abundant miRNA families. IGV browser views of non-chimeric (navy blue) and chimeric (orange and teal) AGO2 binding in the **(A)** cytoplasm and **(B)** nucleus (NLS-AGO2 whole cell). Peak height is defined as read density in reads per million (RPM). All AGO2 peaks are reproducible and meet the following criteria: log2FC>3 (IP vs. input) at least 3 reads per peak. **(C)** Relative level of mature ZFP36 mRNA in DROSHA-/- cells transfected with miRNA mimics and controls. Values are plotted as the average of biological replicates.

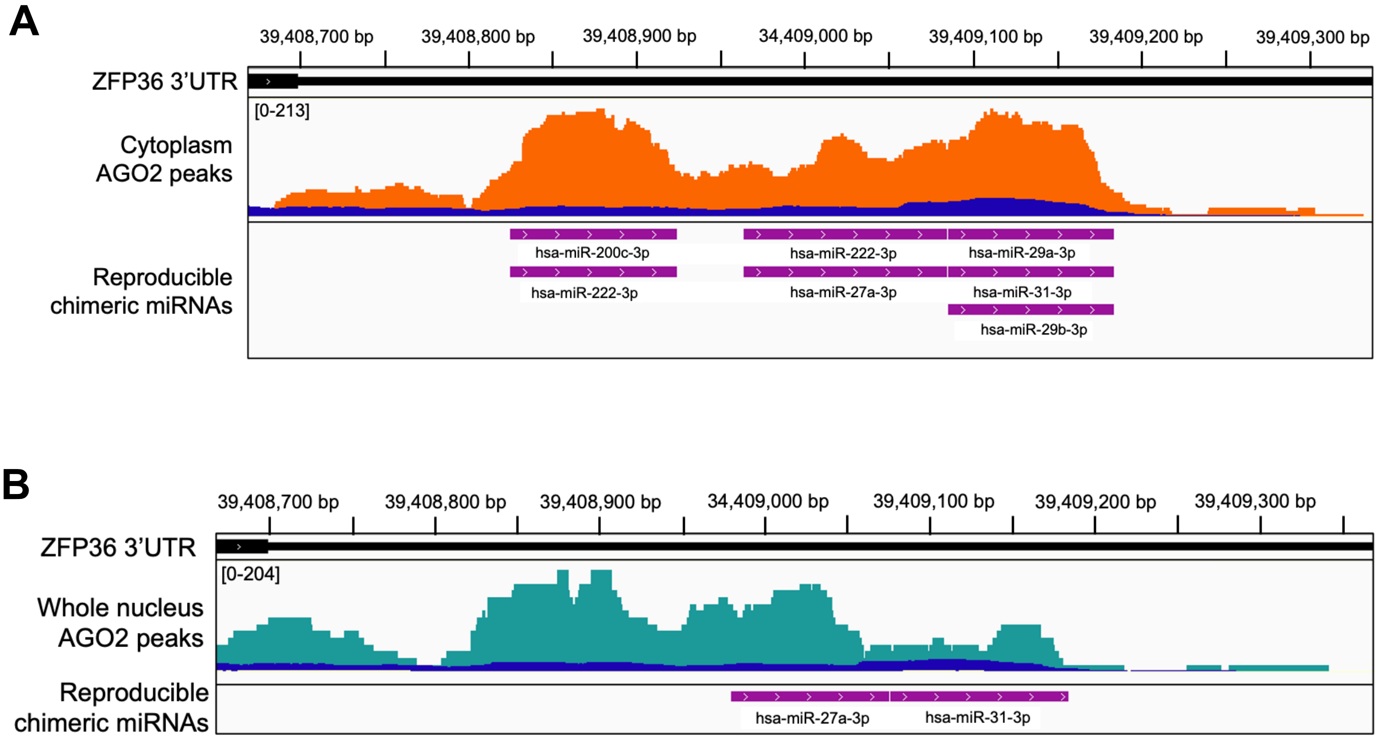

Supplementary figure 14. *ZFP36* is bound by AGO2 in all compartments of the cell. Replicate IGV browser images of chimeric eCLIP reads within the 3'UTR of *ZFP36* in the **(A)** cytoplasm and **(B)** nucleus (NLS-AGO2 whole cell). Chimeric AGO2 peaks are in orange (cytoplasm) and teal (nucleus) and non-chimeric AGO2 peaks are overlayed in navy blue. Peak height is defined as read density in reads per million (RPM). All AGO2 peaks are reproducible and meet the following criteria: log2FC>3 (IP vs. input) at least 3 reads per peak.

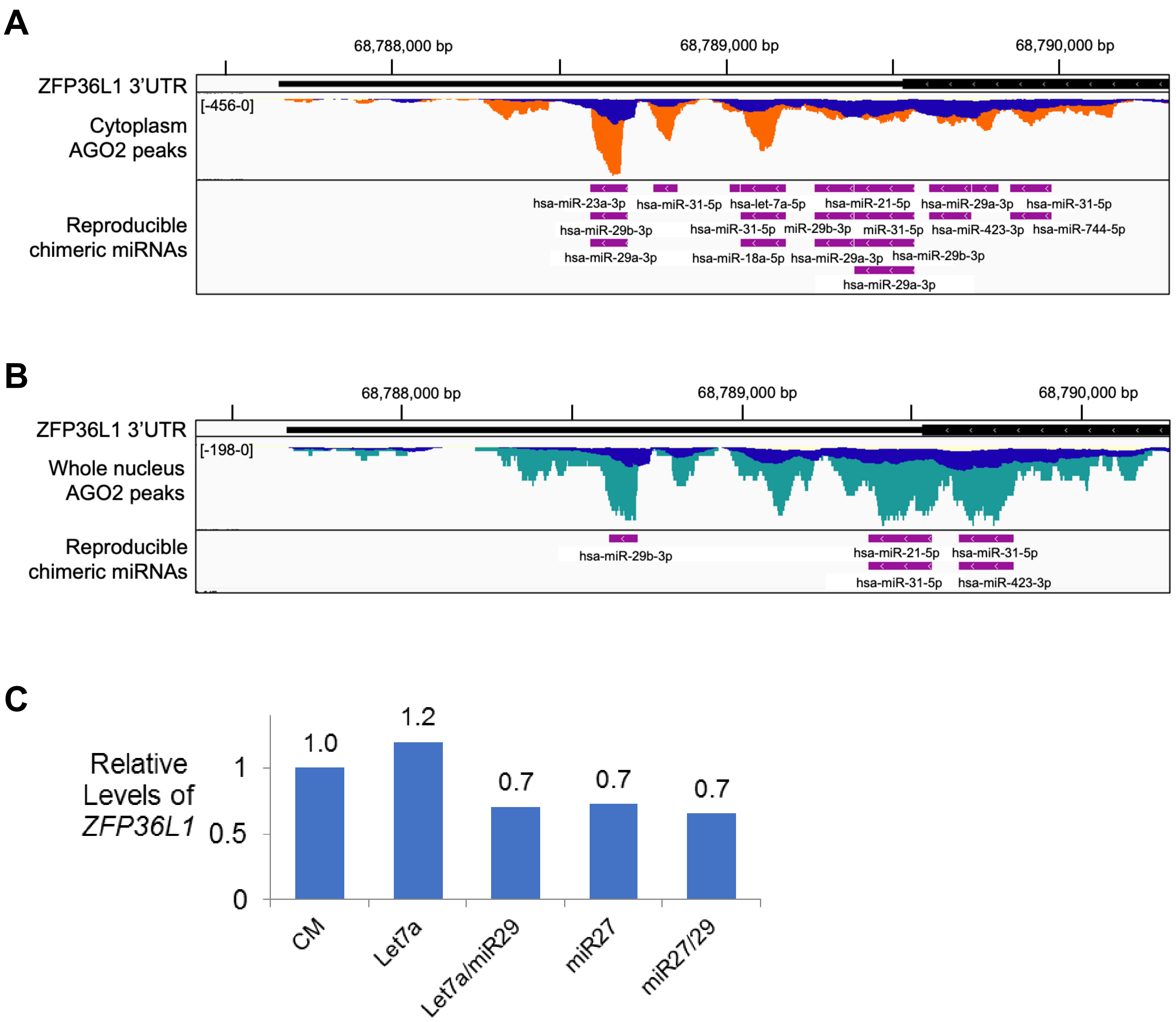

Supplementary figure 15. *ZFP36L1* does not seem to be simply regulated by abundant miRNA families. IGV browser views of non-chimeric (navy blue) and chimeric (orange and teal) AGO2 binding in the **(A)** cytoplasm and **(B)** nucleus (NLS-AGO2 whole cell). Peak height is defined as read density in reads per million (RPM). All AGO2 peaks are reproducible and meet the following criteria: log2FC>3 (IP vs. input) at least 3 reads per peak. **(C)** Relative level of mature ZFP36L1 mRNA in *DROSHA-/-* cells transfected with miRNA mimics and controls. Values are plotted as the average of biological replicates.

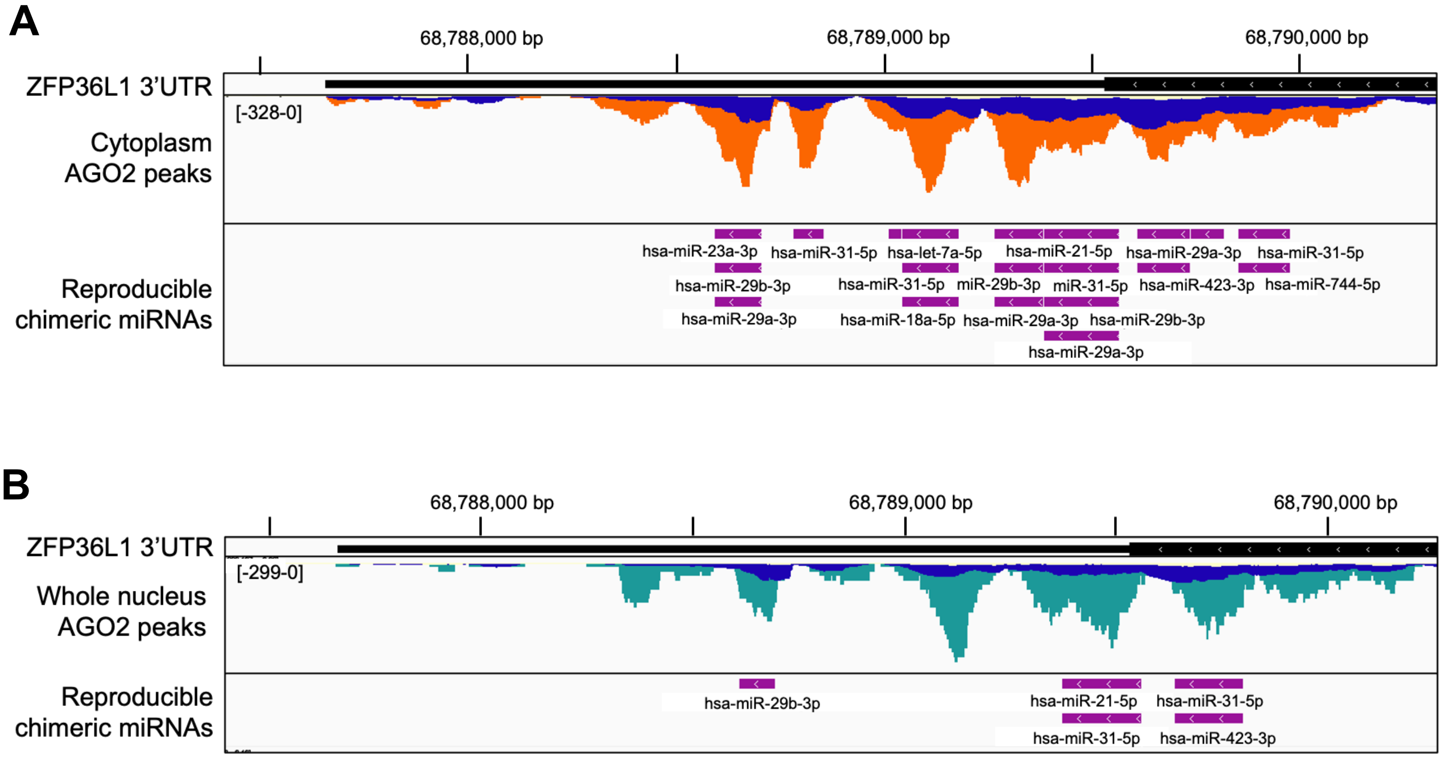

Supplementary figure 16. *ZFP36L1* is bound by AGO2 in cytoplasm and nucleus of HCT116 cells. Replicate IGV browser images of chimeric eCLIP reads within the 3'UTR of ZFP36L1 in the **(A)** cytoplasm and **(B)** nucleus (NLS-AGO2 whole cell). Chimeric AGO2 peaks are in orange (cytoplasm) and teal (nucleus) and non-chimeric AGO2 peaks are overlayed in navy blue. Peak height is defined as read density in reads per million (RPM). All AGO2 peaks are reproducible and meet the following criteria: log2FC>3 (IP vs. input) at least 3 reads per peak.

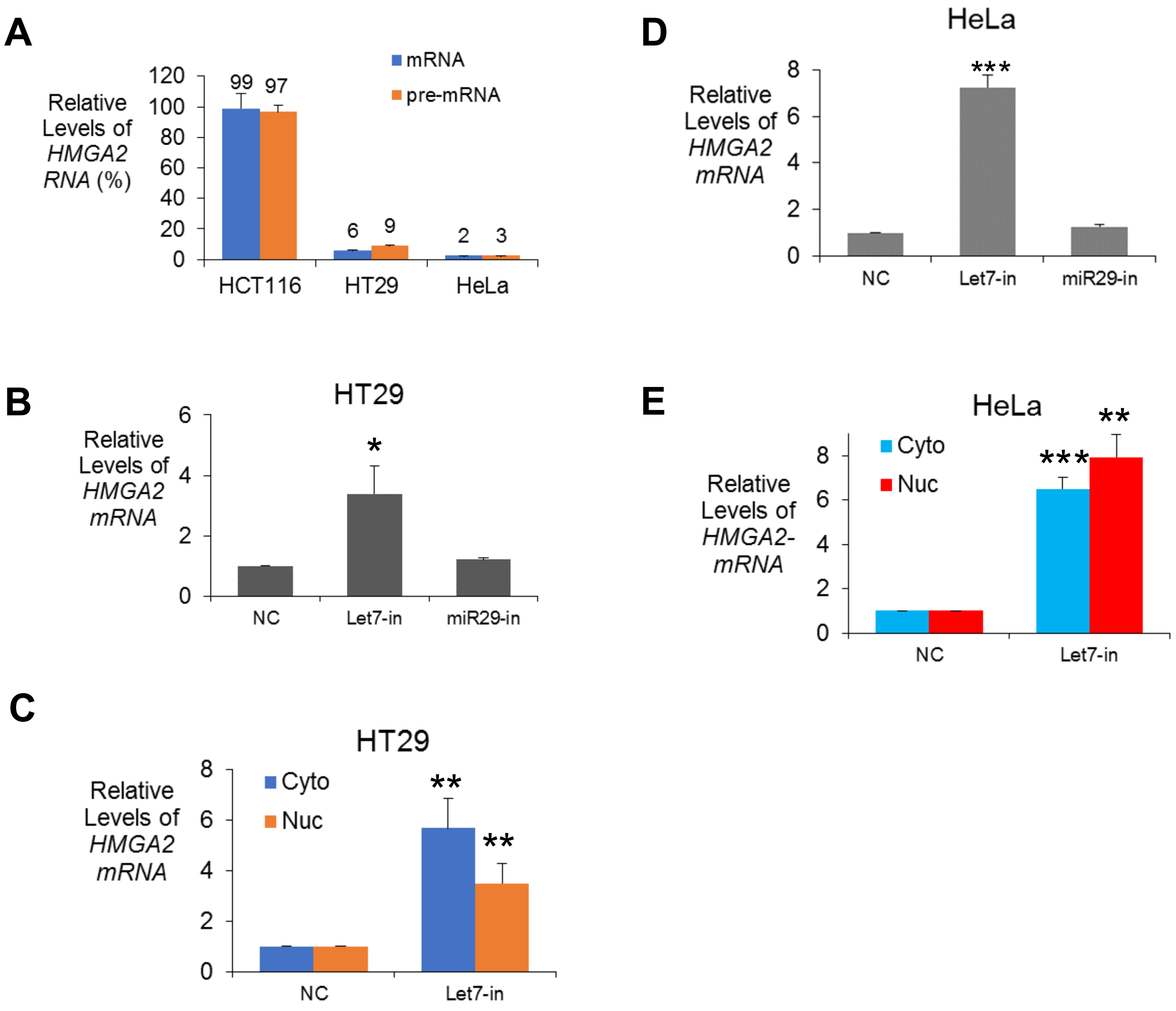

Supplementary figure 17. HMGA2 is silenced by *let-7* family miRNAs in other cell lines. **(A)** Relative level of mature HMGA2 mRNA in HCT116, HT29, and HeLa cells. Relative level of HMGA2 mature mRNA in **(B,D)** whole cell, **(C,E)** cytoplasm, and nucleus following transfection of let-7 family miRNA inhibitor and control miRNA inhibitors in **(B,C)** HT29 and **(D,E)** HeLa cells. Values are plotted as the average +/- SD. Significance denoted as *p<0.05; **p<0.01; ***p<0.001.

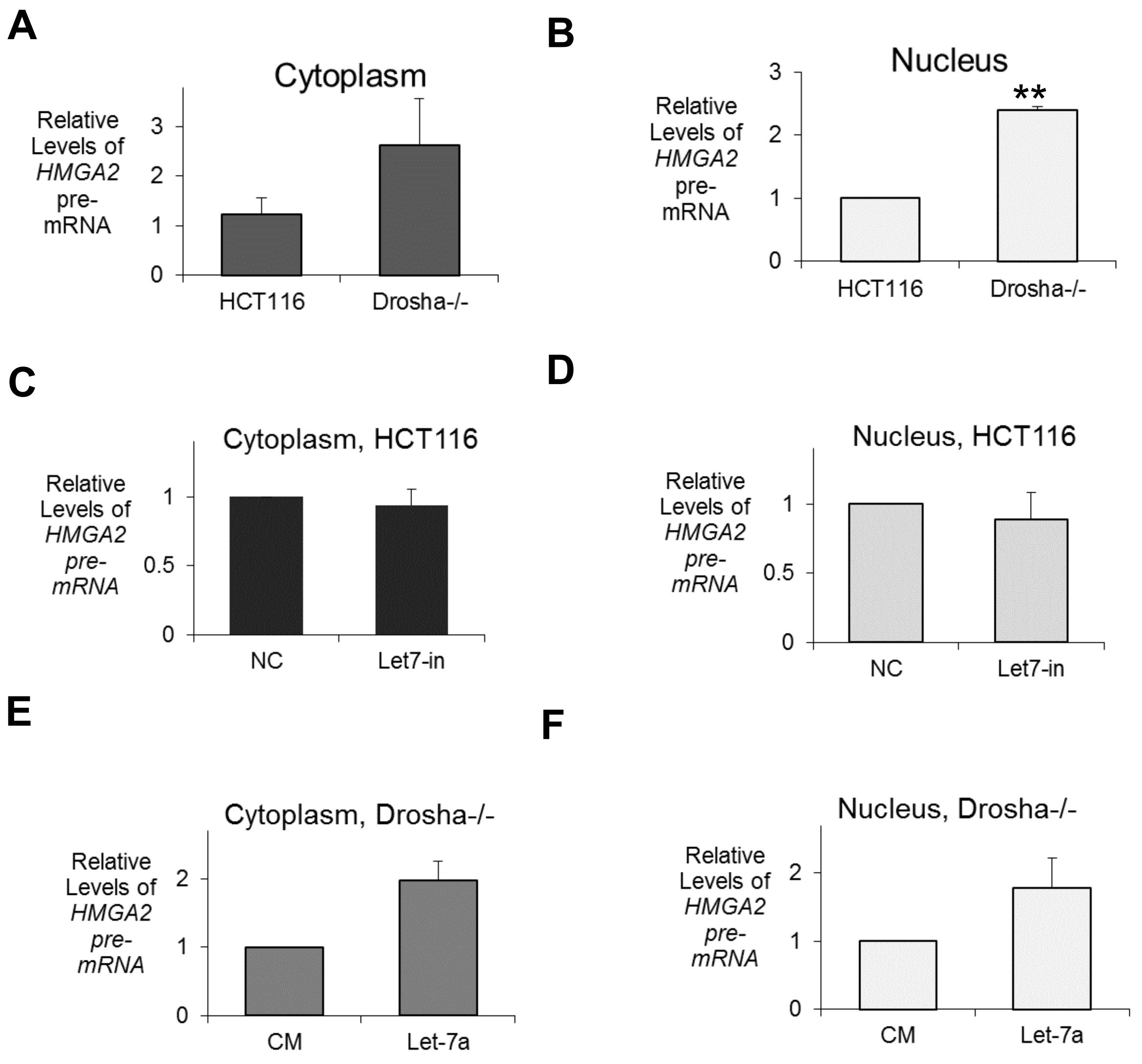

Supplementary figure 18. Impact of *let-7* family miRNA on HMGA2 pre-mRNA levels. Relative level of HMGA2 pre-mRNA in the cytoplasm and nucleus of **(A,B)** untreated WT and Drosha-/-, **(C,D)** WT cells transfected with *let-7* family miRNA inhibitor and **(E,F)** Drosha-/- cells transfected with *let-7a* miRNA mimics. Values are plotted as the average of biological replicates +/-SD. Significance denoted as *p<0.05; **p<0.01; ***p<0.001.

A

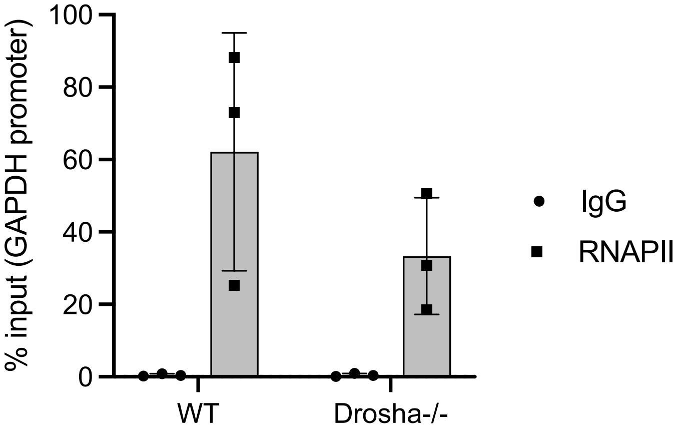

Supplementary figure 19. HMGA2 is not regulated at the level of transcription. ChIP-qPCR results from the **(A)** GAPDH promoter plotted as percent input in both WT and Drosha-/- cells. Points are plotted as the mean of biological replicated +/- standard deviation.

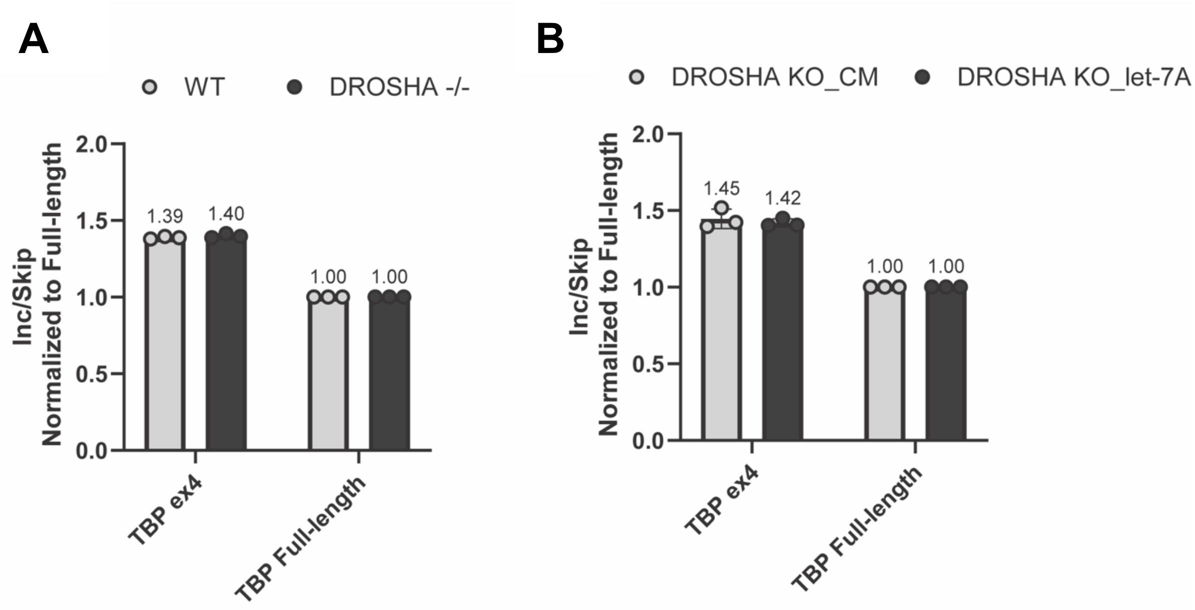

Supplementary figure 20. *Let-7* family miRNAs do not significantly affect splicing of a reference control gene, *TBP*. The RT-qPCR results from this assay, designed similarly to the *HMGA2* splicing assay, are plotted as splicing inclusion:skipping ratios, as shown in (**A)** for untreated WT and DROSHA-/- and (**B)** for DROSHA-/- transfected with *let-7a* miRNA mimic or control. Each condition's respective mean value, calculated from independent biological replicates, are displayed above each condition shown.
